# Acute brain dysfunction clusters in COVID-19: a pilot machine learning-based analysis of the COVID-D cohort

**DOI:** 10.1186/s40635-026-00922-4

**Published:** 2026-06-08

**Authors:** Nekane Romero-García, Víctor Montosa-i-Micó, David Fernández-Narro, Juan M. García-Gómez, Luis Hurtado, Eduardo Passariello, Fabio Silvio Taccone, Chiara Robba, Rameela Raman, Onur M. Orun, Pratik Pandharipande, Brenda T. Pun, E. Wesley Ely, Rafael Badenes, Carlos Sáez

**Affiliations:** 1 Department of Anesthesiology and Critical Care, Hospital Clínico Universitario de Valencia, Avda. Blasco Ibáñez 17, 46010 Valencia, Spain; 2 INCLIVA Research Institute, Avda Menéndez y Pelayo, 4 Accesorio, 46010 Valencia, Spain; 3 Department of Surgery, Faculty of Medicine, University of Valencia, Avda. Blasco Ibáñez 15, 46010 Valencia, Spain; 4 Instituto Universitario de Tecnologías de la Información y Comunicaciones (ITACA), BDSLab, Universitat Politècnica de València, 46022 València, Spain; 5 Service des Soins Intensifs, Hôpital Universitaire de Bruxelles, Hôpital Erasme, Université Libre de Bruxelles, Brussels, Belgium; 6 Department of Surgical Sciences and Integrated Diagnostics, University of Genoa, Genoa, Italy; 7 Anesthesia and Critical Care, San Martino Policlinico Hospital, IRCCS for Oncology and Neuroscience, Genoa, Italy; 8 Critical Illness, Brain Dysfunction, and Survivorship (CIBS) Center, Vanderbilt University Medical Center, Nashville, USA; 9 Division of Anesthesia Critical Care Medicine, Department of Anesthesiology, Vanderbilt University Medical Center, Nashville, TN USA; 10 Department of Biostatistics, Vanderbilt University School of Medicine, Nashville, TN USA; 11 Division of Allergy Pulmonary Critical Care Medicine, Department of Medicine, Vanderbilt University Medical Center, Nashville, TN USA; 12 The Veteran’s Affairs Tennessee Valley Geriatric Research Education Clinical Center (GRECC), Nashville, TN USA

**Keywords:** Acute brain dysfunction, Delirium, Coma, COVID-19, Machine learning, Clusters

## Abstract

**Purpose:**

While acute brain dysfunction (ABD, i.e., delirium and coma) is associated with significantly increased morbidity in critically ill patients, it presents with great heterogeneity that poses a challenge for management and prognostication. While machine learning may be promising for subgroup identification, this approach has not yet been applied to COVID-19 patients with ABD. The aim of our study was to identify distinct clusters among critically ill patients with COVID-19 based on ICU admission data and evaluate their association with clinical outcomes.

**Methods:**

We retrospectively analyzed an international multicenter database (COVID-D study) of critically ill adult patients with COVID-19 during the first pandemic wave and ABD using clinical features on day 1 of admission as input variables. We applied unsupervised machine learning in a pilot attempt to discover clusters of ABD patients. Hierarchical clustering was performed with a bootstrap-based robustness assessment after dimensionality reduction. Clusters were analyzed for differences in neurological outcomes, mechanical ventilation, and survival.

**Results:**

We analyzed 1,631 critically ill COVID-19 patients with ABD, identifying four reproducible clusters with distinct clinical and neurological profiles. Cluster 1 ("mild respiratory failure,” n = 335) had the most favorable outcomes, with the shortest duration of delirium (4.13 days) and mechanical ventilation. Cluster 2 ("moderate ARDS," n = 508) showed a comparable delirium incidence but the longest duration (5.18 days). Cluster 3 ("early severe ARDS," n = 161) included patients who underwent prone positioning and mechanical ventilation early from the day of admission, with higher rates of coma (100%), including persistent coma (27.3%). Cluster 4 ("late severe ARDS," n = 475) represented severely ill patients with the longest coma duration (11.2 days) and the lowest delirium-free and coma-free (DFCF) days (4.74), in relation to deep and prolonged sedation. Despite the wide range of ABD durations across four groups, no significantly different 28-day mortality (23.6–38.0, p > 0.78), ICU (15.8–19.2 days range, p = 0.154) and hospital (22.5–26.7 days range, p = 0.259) length of stay were observed among clusters.

**Conclusion:**

This pilot analysis of ICU admission data from the first COVID-19 wave suggests the existence of clinically distinct clusters among patients with acute brain dysfunction. Differences were observed in the type and duration of delirium and coma, though these did not translate into differences in 28-day survival. This exploratory work may support targeted delirium prevention strategies, but prospective studies are required to determine its clinical utility in modern ICU settings.

**Graphical Abstract:**

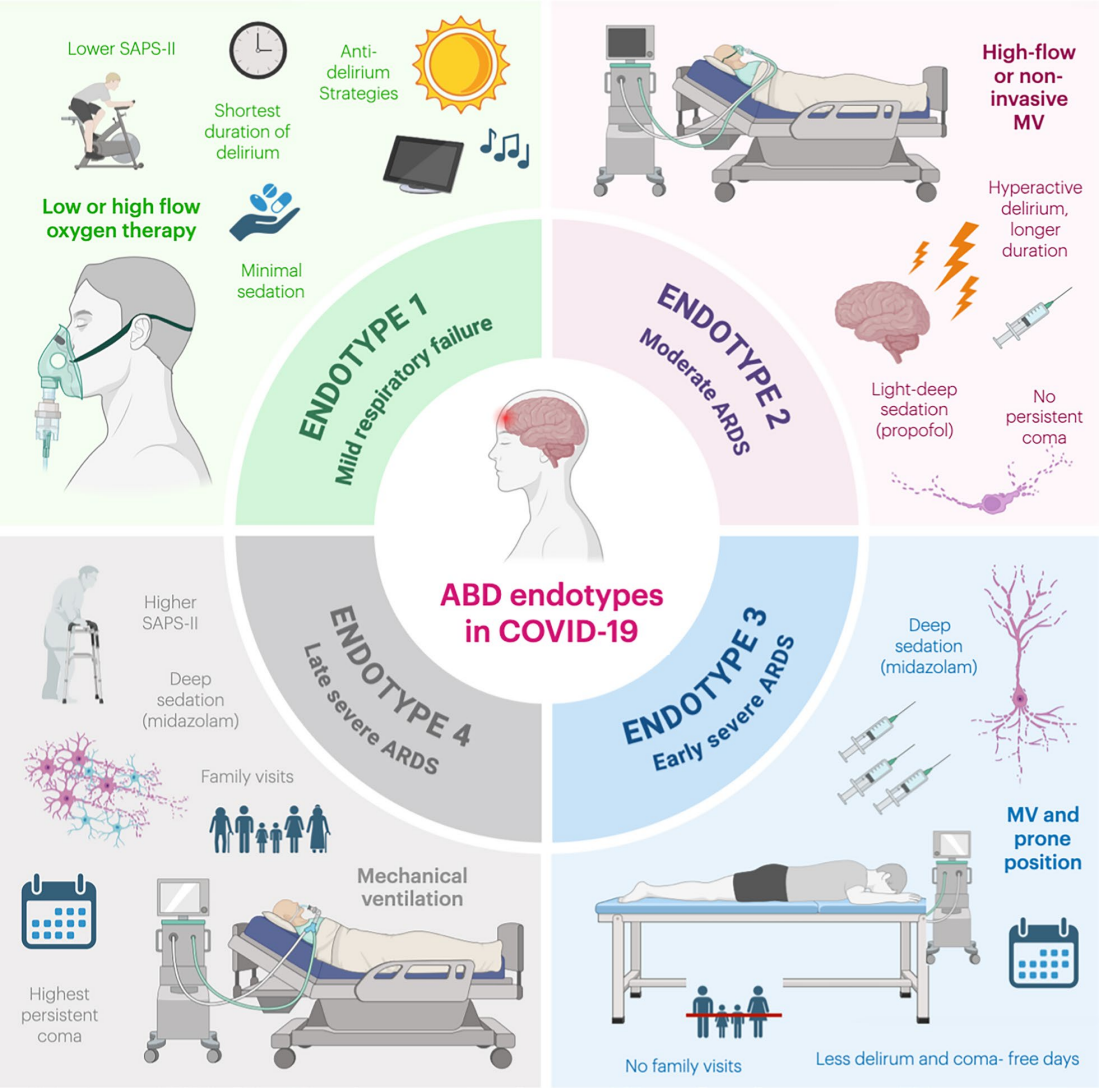

**Supplementary Information:**

The online version contains supplementary material available at 10.1186/s40635-026-00922-4.

## Introduction

Acute brain dysfunction (ABD)—a condition encompassing a clinical spectrum from delirium to coma—is associated with increased morbidity and mortality in critically ill patients [1, 2]. Its clinical and pathophysiological heterogeneity—ranging from hyperactive to hypoactive presentations and driven by factors such as neuroinflammation, hypoxia, metabolic derangements, and drug effects—poses challenges for management and prognostication [3–5]. Responses to interventions, including sedatives and ventilation [6, 7], also vary widely, contributing to the complexity of ABD trajectories, from transient episodes to prolonged coma or lasting cognitive impairment [8, 9]. This variability highlights the need for precision approaches tailored to different patient groups [10].

The implementation of care bundles, including routine delirium monitoring, has been associated to reduced incidence of ABD and improved prognosis [11–13], while prognostic models such as PRE-DELIRIC have shown potential in predicting risk [14]. Machine learning (ML), with its ability to integrate large volumes of data, can uncover complex relationships between variables [15–17], making it especially useful in heterogeneous conditions like ABD where traditional approaches are limited [18, 19]. These insights could guide hypothesis generation and future development of targeted therapies [20, 21].

While ML has shown promise in identifying delirium subgroups, most studies rely on general ICU data instead of disease-specific data [20], and have not yet been applied to COVID-19 cohorts, despite the high prevalence of ABD in this population [22]. COVID-19 presents a distinct clinical context—marked by inflammation, hypoxemia, and prolonged sedation—where traditional ABD models may not apply [23]. Moreover, most previous studies do not consider non-pharmacological measures and sedation practices, which have proven key for delirium prevention [24]. Given the burden of post-COVID neurological manifestations and the costs of ABD, ML-based prognostic tools could offer valuable insights in this setting [25–27].

We hypothesized that identifying data-driven clusters in this setting could be helpful in predicting varied outcome trajectories of patients with ABD [21, 28]. The primary aim of our pilot study was to identify distinct clusters of ABD among critically ill patients with COVID-19 using unsupervised ML. Secondary objectives included evaluating the temporal stability of these clusters and their associations with key clinical outcomes such as duration of brain dysfunction, ventilatory support, and mortality.

## Methods

### Data collection

We performed a secondary analysis of the COVID-D dataset, an observational multicenter cohort study of 69 adult ICUs across 14 countries [23]. Adult admitted to participating ICUs with confirmed severe acute respiratory syndrome coronavirus 2 (SARS-CoV-2) infection from the fist detected case to April 28th, 2020 were included. The COVID-D study excluded patients with pre-existing mental illnesses, neurodegenerative disorders, congenital or acquired brain damage, hepatic coma, prisoners, patients with drug overdose or suicide attempt, or those who had life-support measures withdrawn within 24 h of ICU admission. For the primary analysis, we included only patients who experienced ABD (i.e. delirium or coma) at any time during ICU stay. Patients without ABD were included for exploratory purposes in preliminary analyses. De-identified data were extracted from electronic health records. Most variables collected in the original study on day of admission, including patient demographics and initial treatment, were included as inputs. Variables collected during ICU stay, including delirium or coma assessments, management strategies, with additional data on ventilator support, ICU length of stay, and survival, as well as race, were analyzed as outcomes. A comprehensive description of the variables can be found in the Supplement (Tables S1 and S2). Mental status was defined as follows: coma was defined as a day when the patients were unresponsive to verbal stimulation (RASS equivalent score –4 or –5 or Glasgow Coma Scale score of < 8); patients were considered delirious if they were responsive to verbal stimulation and had a positive delirium assessment scale assessment documented; if a patient was responsive to verbal stimulation but was not delirious, they were considered to be awake without delirium. A positive delirium assessment was defined as: CAM-ICU positive, or ICDSC ≥ 4 points, or DOS ≥ 3 points, or Nu-DESC ≥ 2 points, or 4AT ≥ 4 points. In the original dataset, neurological assessments and management strategies were collected until day 21, discharge or death, whatever happened first; ventilatory support, length of stay and survival were recorded for 28 days [23]. Study data were collected and managed using REDCap electronic data capture tools hosted at Vanderbilt University Medical Center [29, 30].

### Data preprocessing and variable selection

**Fig. 1:**
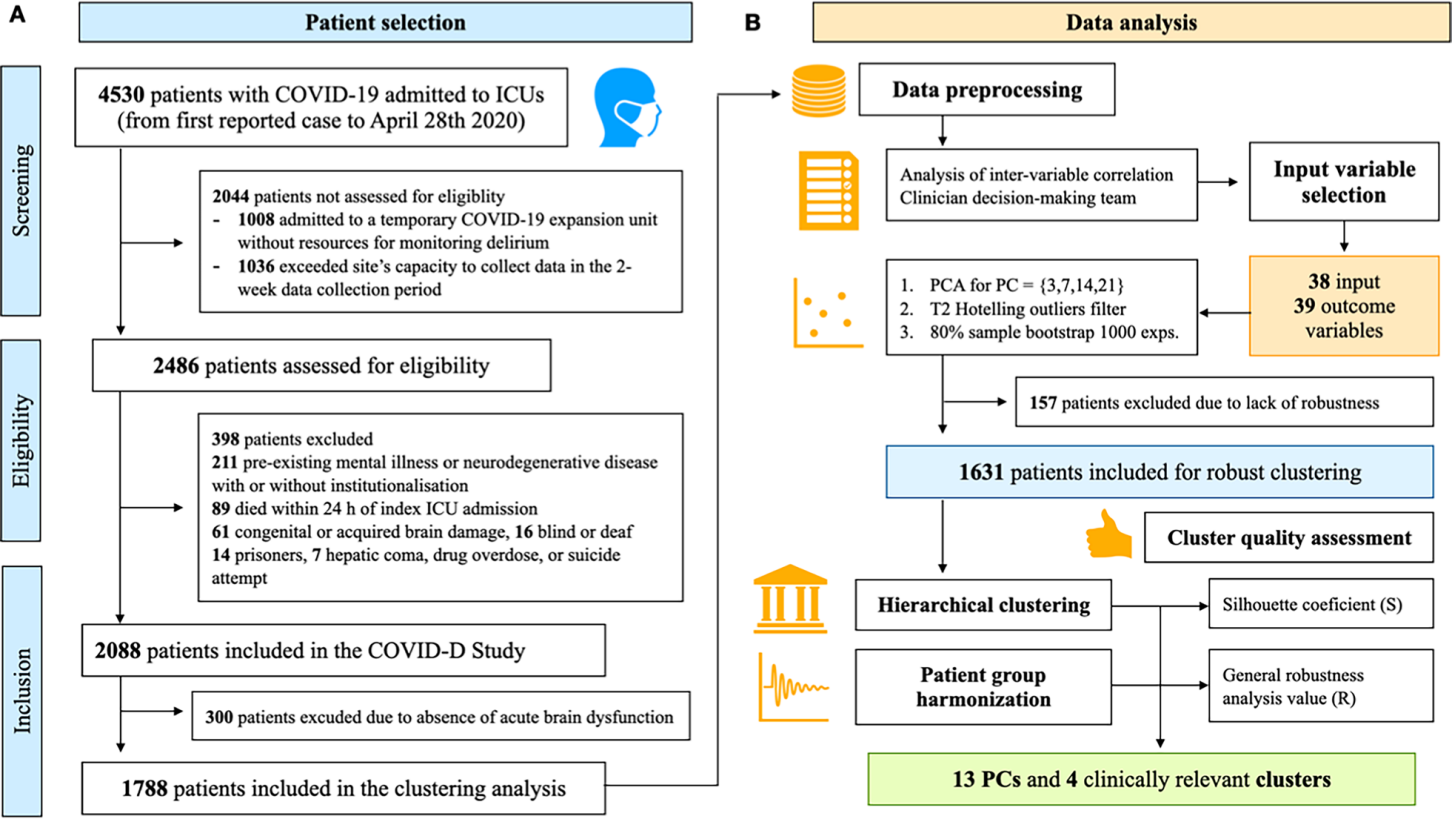
Study flowchart diagram. **A** Study population flowchart from the COVID-D study population to final robust clustering analysis according to CONSORT guidelines. **B** Methodological pipeline from variable selection to preprocessing, hierarchical clustering, group harmonization and cluster quality analysis

We developed a methodological pipeline (Fig. 1) to ensure robust and clinically interpretable clustering outcomes across different parameterizations and real-world data variability [31]. Input variables collected on the first day of ICU admission were selected for clustering analysis to predict clusters; they summarized the patient’s characteristics, previous comorbidities, sedation strategies, and ventilatory and hemodynamic support. Outcome variables, predicted by the inputs, were included in tables to analyze the clinical phenotypes corresponding to each of the identified subgroups. They summarized: (i) prevalence and duration of ABD, including delirium and coma, as well as delirium subtypes, (ii) hospital stay and 28-day survival and (iii) need for mechanical ventilation. The delirium-free and coma-free days (DFCF) were calculated in the COVID-D original study using a computation of the number of days within the 21-day study period where the patient was alive and free of delirium or coma. Categorical variables were binarized prior to principal component analysis (PCA). To ensure the robustness of the clustering analysis and minimize bias, input variables were selected based on data integrity and clinical relevance. First, a complete-case variable selection was applied; only variables with 100% completeness across the entire cohort were included, thus avoiding the need for imputation and preventing patient-level exclusion. Second, a correlation analysis was conducted to identify redundant information; in cases of high collinearity, the most clinically significant variable was retained (see Statistical Analysis in the Supplement).

### Clustering methodology and cluster quality assessment

The clustering methodology used is based on the study and combination of different parameters [32, 33]. Hierarchical clustering was performed on PCA-reduced data, following the exclusion of outliers with Hotelling’s T-squared distribution. To ensure robustness, clustering was repeated on 1000 bootstrap samples and consistency across runs was harmonized using the Hungarian algorithm [34]. Final cluster assignment was based on the most frequently assigned group per patient. Cluster quality was assessed using the robustness index (R) and Silhouette Mean Index (S). Heatmaps of R and S across PCA variance levels and k values guided performance evaluation. Robustness of individual assignments was tested using a binomial approach (see Statistical Analysis in the Supplement).

### Visualization of results

Scatter plots are used to depict the inter- and intra-group associations among robust patients. Outcome variable distribution within clusters are presented in tables. We used chi-square tests for categorical variables and ANOVA for continuous variables to assess whether the differences between the groups were statistically significant. The primary outcome of this analysis are DCDFs, and secondary outcomes are incidence of delirium and coma, delirium subtypes, persistent coma, mortality, LOS and need for mechanical ventilation. All statistical analyses and machine learning clustering were performed using R software version 4.4.1 (R Foundation for Statistical Computing, Vienna, Austria).

## Results

### Study population and variables selection

Between January 20th and April 28th, 2020, 4,530 patients admitted to ICU with COVID-19 were screened, from which 2,088 were finally enrolled in the COVID-D cohort. From these, 1,788 patients (85.6%) experienced ABD during their ICU stay and were included in our analysis. After cluster quality assessment and outliers removal, the final sample size was reduced to 1,631 patients, representing 78.1% of the patients in the original COVID-D cohort (Fig. 1). For exploratory purposes, we performed a preliminary analysis including patients without ABD, which included 1,875 patients after cluster quality assessment and outliers removal (Table S6, S7 and Figure S2 in the Supplement).

**Fig. 2:**
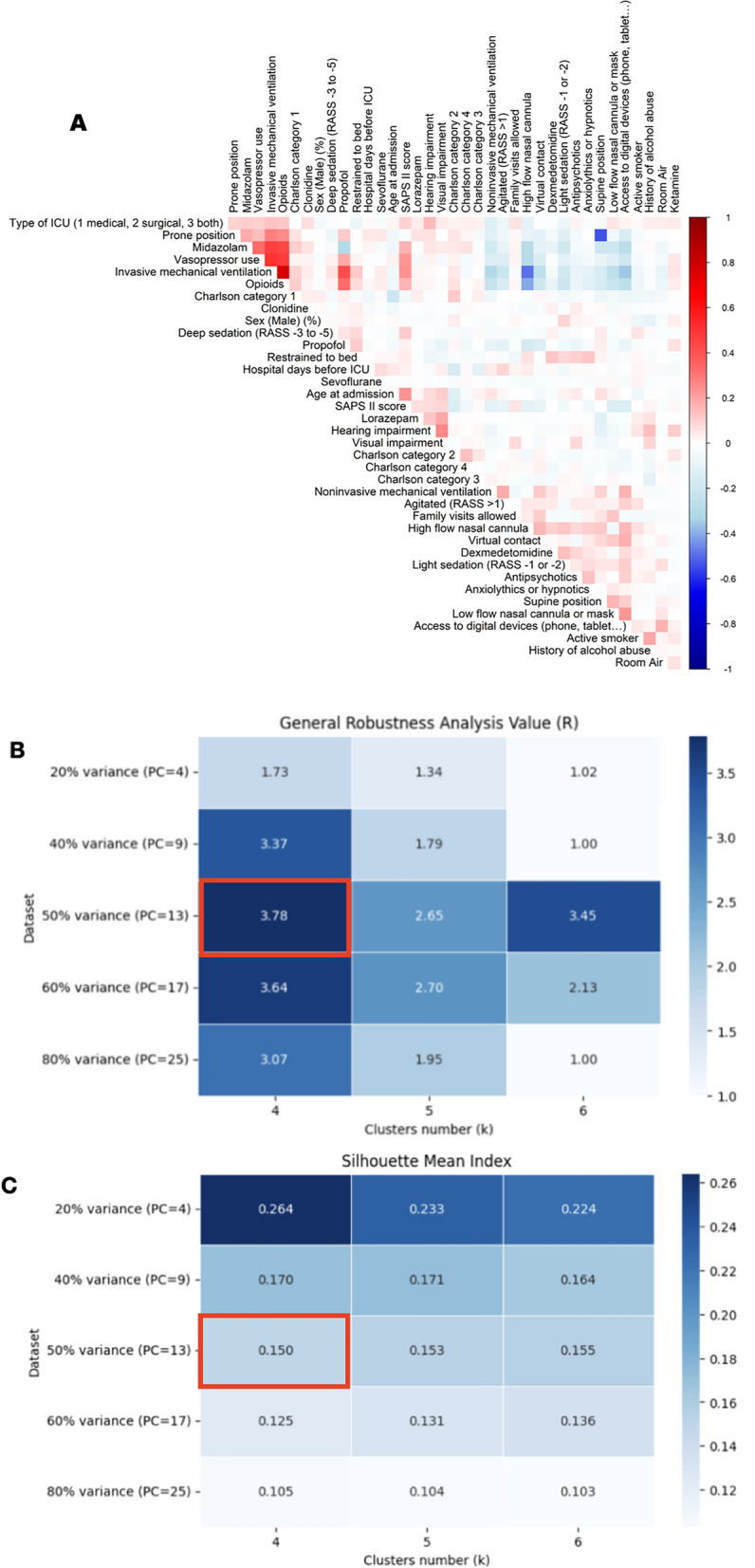
Selection of variables, optimum number of clusters and principal components. **A** Linear correlation between pairs of variables. To visualize the strength of linear correlation between each pair of features, the value of the Pearson correlation coefficient is represented by the size and colour of the dots in the matrix. **B** General robustness analysis value (R) for each number of clusters (k) and each amount of variance. **C** Mean Silhouette value (S) for each number of clusters (k) and each amount of variance. Red box represents the number of principal components and clusters chosen for the analysis. PC: number of Principal Components

A total of 38 variables were selected as inputs for clustering, after discarding those with missing values or high correlation with other variables (Fig. 2A); 39 variables were considered as outcome measures, representing neurological outcomes, mechanical ventilation requirements and survival. The average age of the patients was 62.3 $\pm$ 11.9 years, with 27.9% being male. The mean SAPS II score at ICU admission was 44.6 $\pm$ 16.4. Overall, 33.2% of patients died, while 96.3% experienced coma, with a mean duration of 10.1 days (SD = 6.04). Delirium was observed in 63.8% of patients, lasting on average 4.5 days (SD = 3.48). Notably, most patients (97.3%) required mechanical ventilation (Table 1, Table S1 and S2 in the Supplement).

**Table 1:** Distribution of most clinically relevant input variables in ABD clusters in the COVID-D database

	Cluster 1 (n = 335)	Cluster 2 (n = 508)	Cluster 3 (n = 161)	Cluster 4 (n = 475)	p value
Patients’ characteristics (%|x, CI 95%)					
Age at admission	63.14 (61.84–64.45)	62.42 (61.41–63.43)	59.88 (57.94–61.81)	62.67 (61.59–63.75)	0.034***
Sex (Male) (%)	27.46 (22.68–32.24)	29.53 (25.56–33.49)	31.06 (23.91–38.2)	29.05 (24.97–33.14)	0.8544
Hearing impairment	4.18 (2.04–6.32)	0 (0–0)	0.62 (-0.59–1.83)	6.74 (4.48–8.99)	0***
Visual impairment	0.9 (-0.11–1.9)	0 (0–0)	0 (0–0)	2.53 (1.12–3.94)	5e-04***
SAPS II score	36.93 (35.42–38.44)	44.31 (43.01–45.61)	44.47 (41.94–46.99)	49.42 (47.84–51.01)	0***
Active smoker	9.85 (6.66–13.04)	5.71 (3.69–7.73)	6.83 (2.94–10.73)	1.89 (0.67–3.12)	0***
History of alcohol abuse	3.28 (1.38–5.19)	1.77 (0.62–2.92)	4.35 (1.2–7.5)	0.84 (0.02–1.66)	0.0171***
Charlson category 1	52.24 (46.89–57.59)	59.65 (55.38–63.91)	69.57 (62.46–76.67)	68 (63.81–72.19)	0***
Type of ICU (1 medical, 2 surgical, 3 both)	1.87 (1.76–1.98)	1.99 (1.9–2.07)	2.09 (1.96–2.23)	2.29 (2.21–2.37)	0***
Ventilatory support on day 1 (%|x, CI 95%)					
Low flow nasal cannula or mask	44.18 (38.86–49.5)	19.09 (15.68–22.51)	2.48 (0.08–4.89)	15.37 (12.13–18.61)	0***
High Flow nasal cannula	49.55 (44.2–54.91)	10.24 (7.6–12.87)	1.24 (-0.47–2.95)	2.74 (1.27–4.2)	0***
Noninvasive mechanical ventilation	23.28 (18.76–27.81)	7.68 (5.36–9.99)	0.62 (-0.59–1.83)	0.21 (-0.2–0.62)	0***
Invasive mechanical ventilation	2.09 (0.56–3.62)	98.43 (97.34–99.51)	99.38 (98.17–100.59)	100 (100–100)	0***
Prone position	5.07 (2.72–7.42)	27.95 (24.05–31.86)	100 (100–100)	26.95 (22.96–30.94)	0***
Vasopressor use	4.78 (2.49–7.06)	62.2 (57.99–66.42)	68.32 (61.14–75.51)	74.32 (70.39–78.24)	0***
Medications on day 1 (%|x, CI 95%)					
Opioids	4.48 (2.26–6.69)	86.81 (83.87–89.75)	95.03 (91.67–98.39)	94.11 (91.99–96.22)	0***
Antipsychotics	0 (0–0)	0.98 (0.13–1.84)	0 (0–0)	0 (0–0)	0.0224***
Anxiolytics or hypnotics	11.34 (7.95–14.74)	5.51 (3.53–7.5)	6.21 (2.48–9.94)	2.95 (1.43–4.47)	0***
Sedation drugs on day 1 (%|x, CI 95%)					
Propofol	0.9 (-0.11–1.9)	91.34 (88.89–93.78)	35.4 (28.02–42.79)	15.79 (12.51–19.07)	0***
Midazolam	0.3 (-0.29–0.88)	15.55 (12.4–18.7)	77.64 (71.2–84.08)	92 (89.56–94.44)	0***
Dexmedetomidine	1.49 (0.19–2.79)	5.12 (3.2–7.03)	0.62 (-0.59–1.83)	0 (0–0)	0***
Anti delirium measures on day 1 (%|x, CI 95%)					
Restrained to bed	7.16 (4.4–9.93)	25.98 (22.17–29.8)	16.15 (10.46–21.83)	11.16 (8.33–13.99)	0***
Mobility or exercise (active range of motion, sitting, standing, walking)	33.43 (28.38–38.48)	10.63 (7.95–13.31)	1.86 (-0.23–3.95)	1.89 (0.67–3.12)	0***
In-person visits (family/friends)	6.57 (3.91–9.22)	0.2 (-0.19–0.58)	2.48 (0.08–4.89)	14.11 (10.98–17.24)	0***
Virtual contact (via digital devices, e.g. phones or tablets)	20.3 (15.99–24.61)	1.77 (0.62–2.92)	0.62 (-0.59–1.83)	5.05 (3.08–7.02)	0***
Sedation level on day 1(%|x, CI 95%)					
No sedation (RASS 0)^†^	81.79 (77.66–85.92)	3.74 (2.09–5.39)	0.62 (-0.59–1.83)	2.53 (1.12–3.94)	
Light sedation (RASS -1 or -2)	12.24 (8.73–15.75)	7.28 (5.02–9.54)	0 (0–0)	0 (0–0)	0***
Deep sedation (RASS -3 to -4)	1.19 (0.03–2.36)	6.3 (4.19–8.41)	0.62 (-0.59–1.83)	1.05 (0.13–1.97)	0***
Deep sedation (RASS -5)^†^	1.49 (0.19–2.79)	82.28 (78.96–85.6)	98.76 (97.05–100.47)	96.42 (94.75–98.09)	0***
Agitated (RASS > 0)	3.28 (1.38–5.19)	0.39 (-0.15–0.94)	0 (0–0)	0 (0–0)	0***

Dichotomic variables are represented as relative frequencies of value = 1 (0 = “no” and 1 = “yes”). For ICU type, 1 = medical ICU, 2 = surgical ICU, 3 = mixed ICU. *p < 0.05, **p < 0.01, ***p < 0.001

^†^Variables “no sedation” and “coma” were excluded from the analysis due to high correlation with other variables; however, they are shown here for better interpretation of clusters

### Choice of optimum number of clusters (k) and principal components (PC)

The decision regarding the amount of variability to retain and the optimal number of clusters to select for presenting the clinical results was based on General Robustness Analysis Value (*R*) for each clustering configuration (Fig. 2B). Given that the values in each column (corresponding to the number of clusters) range from 1 to *k*, it is evident that the values for *k* = 4 are significantly higher both absolutely and relatively, with the highest value of 3.78 observed when 50% of the variance is retained. Figure 2C presents the Silhouette Mean Index (*S*), revealing that the number of principal components analyzed is the key factor influencing the silhouette score. A smaller number of variables leads to better silhouette values. However, for cluster identification, we selected the configuration with *k* = 4 and 50% of variance retained, as despite achieving a lower silhouette score compared to lower-dimensional settings, we prioritized the robustness of the results according to *R*, ensuring a better representation of the data structure while preserving a greater amount of information.

### Importance of variables included in the model

**Fig. 3:**
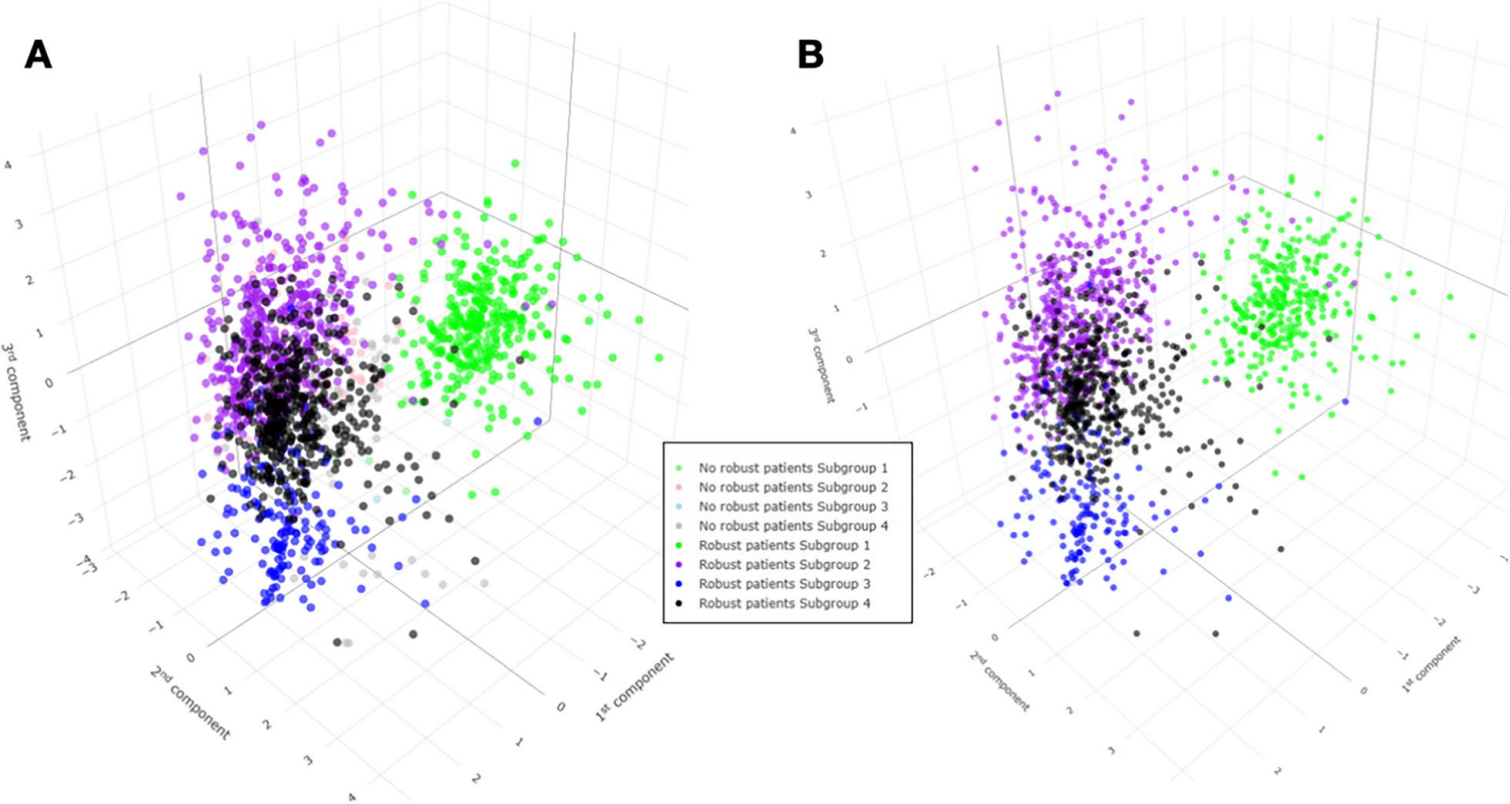
Three-dimensional PCA plot for cluster distribution. **A** 3D Scatterplot for all patients. Dark-colored dots represent robust patients, while light-colored dots represent non-robust patients. **B** 3D Scatterplots for robust patients only. This visualization enables the analysis of both intragroup and intergroup differences, providing valuable insights into the structure and heterogeneity of the identified clusters. A clear separation of cluster 1 is observed, whereas cluster 4 overlaps with cluster 2 and 3, suggesting a greater similarity. Additionally, patients classified as non-robust according to the binomial test tend to deviate from the center of their respective clusters, reinforcing the idea that they do not fully conform to their assigned cluster structure

For 13 principal components and 4 clusters, the PCA plot shows a clear separation of cluster 1, while cluster 4 overlaps with clusters 2 and 3, indicating greater similarity between them (Fig. 3). Patients’ characteristics (age at admission, sex, hearing or visual impairment), previous disease history (including SAPS II score, active smoker status, history of alcohol abuse, and Charlson comorbidity categories 1–4), ICU admission details (type of ICU, days in hospital before ICU admission), types of ventilatory support (room air, low-flow nasal cannula or mask, high-flow nasal cannula, noninvasive mechanical ventilation, invasive mechanical ventilation, supine or prone position) and hemodynamic support (vasopressor use) received on day of admission, sedation practices on admission (use of opioids, antipsychotics, anxiolytics or hypnotics, and specific drugs such as propofol, midazolam, dexmedetomidine, sevoflurane, lorazepam, clonidine, and ketamine) and sedation levels (light, deep, or agitated sedation based on RASS scores), and anti-delirium measures (restraint use, access to digital devices, family visits, virtual contact), were identified as the most important features in our model. In practice, this comprised all variables collected on day 1, with the exception of: specific opioids, antipsychotics, and other medications (including dosage), due to a high proportion of missing data; detailed agitation/sedation scales, given their high correlation with the general 'sedation level on day 1' variable, which integrated this information; and race, which was excluded from the clustering input and instead included in outcome models to adjust for potential confounding.

### Clusters characteristics

**Fig. 4:**
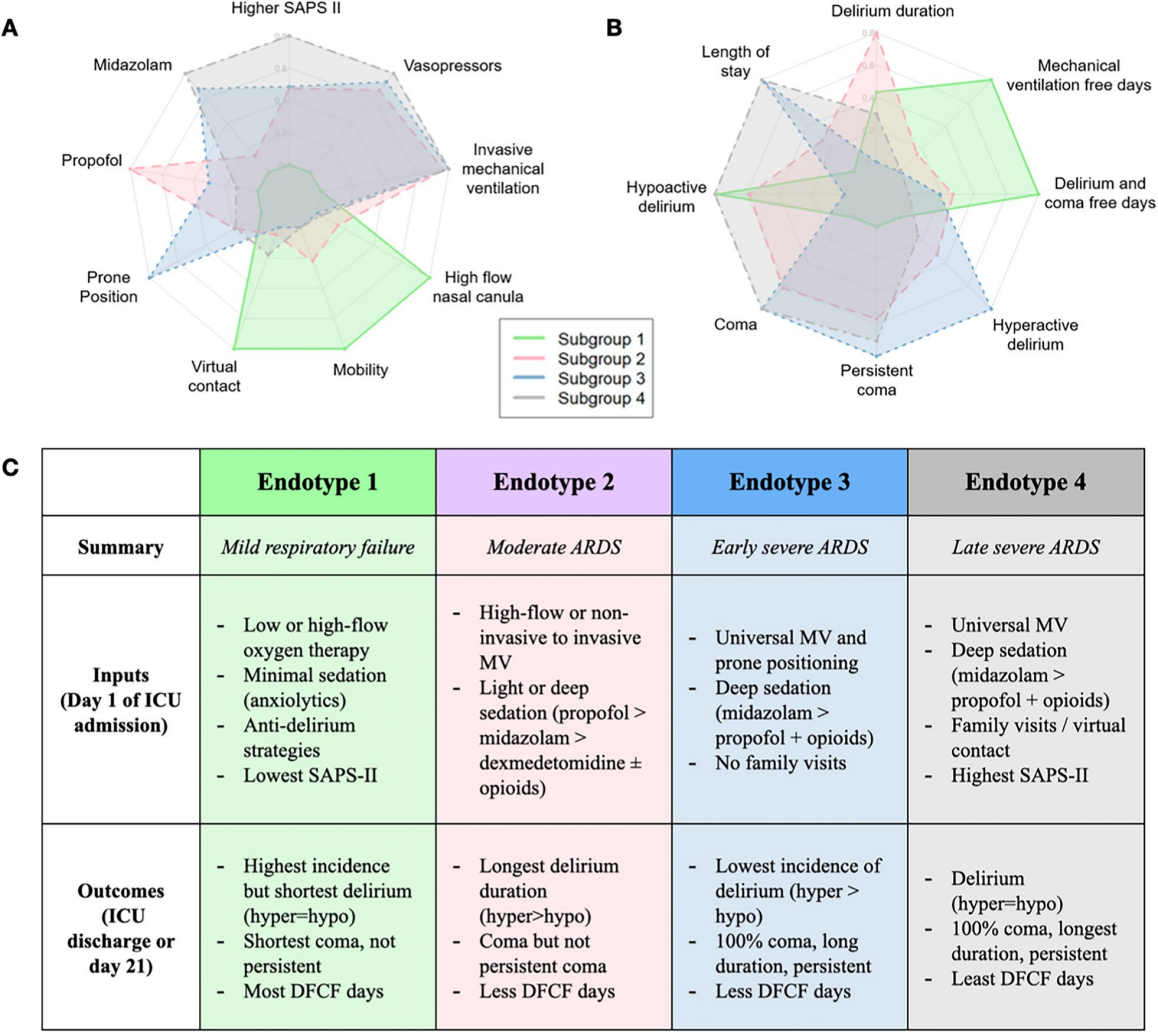
Clinical summary of ABD clusters. **A** Radar plot for distribution of clinically relevant input variables at admission across robust clusters. **B** Radar plot for distribution of clinically relevant outcomes across robust clusters. **C** Summary of results. ARDS: Acute respiratory distress syndrome; DFCF days: Delirium-free and coma-free days, MV: Mechanical ventilation, SAPS-II: Simplified Acute Physiology Score II. “ > ” represents “more than”

Median values for input and output variables in the different clusters are presented in Table 1 and in Supplementary Tables S4 and S5. A visual description of cluster characteristics is provided Fig. 4A. Cluster 1 (“mild respiratory failure,” n = 335) represents less severe patients who were awake under mild support measures. It is characterized by a predominant use of non-invasive respiratory support, with 49.6% of patients receiving high-flow nasal cannula and only 2.1% requiring invasive mechanical ventilation. Most patients were awake (81.8%) or under light sedation (12.2%) on day 1 according to RASS, although some were agitated (3.2%). Most frequently used sedative drugs were anxiolytics or hypnotics (11.34%), with a minimal proportion of opioids (4.5%), dexmedetomidine (1.5%) and propofol (0.9%). Anti-delirium strategies were more available compared to other clusters: 33.4% of patients had documented exercise or mobility on that day (e.g. active range of movement, sitting on edge of bed, standing, or walking), 20.3% maintained virtual contact with family members, and 6.6% had in-person family visits. This group includes the highest proportion of patients with smoking or alcohol abuse history, and it includes patients with cardiogenic shock or myocardial infarction (n = 2).

Cluster 2 (“moderate ARDS,” n = 508) includes patients with moderate severity of disease who experienced clinical worsening during the first day of ICU admission and required invasive mechanical ventilation. Various forms of non-invasive ventilatory support were used, including low-flow oxygen (19.1%), high-flow cannulas or masks (10.2%), and non-invasive ventilation (7.7%). Invasive mechanical ventilation was used in the majority of these patients on day 1 (98.4%), with prone positioning applied in a moderate proportion (27.9%). Notably, most patients in this group were sedated with propofol (91.3%), followed by midazolam (15.6%); the use of dexmedetomidine was the highest in this cluster (5.1%), and opioid use increased in great extent (86.8%). There is a decrease in anti-delirium measures compared to cluster 1, with less family visits or virtual contact, and the highest rates of physical restraint use (25.9%), probably due to respiratory worsening. Early hemodynamic instability was evident, with 62.2% requiring vasopressors. The lowest RASS score, reflecting the maximum sedation level, was − 4 (6.3%) or − 5 (82.2%). This group also includes a minority of patients admitted due to other conditions, such as cardiac arrest (n = 1), traumatic brain injury (n = 1) or seizures (n = 2).

Cluster 3 (“early severe ARDS,” n = 161) is defined by a critical respiratory profile, with 100% of patients being admitted due to a SARS-CoV-2 diagnosis. All patients (100%) underwent prone positioning, with nearly universal invasive mechanical ventilation (99.4%) and intensive sedation to RASS − 5 (98.8%). Sedation patterns shifted away from propofol dominance as only 35.4% received propofol, while a large proportion (77.6%) were treated with midazolam, and opioid use was higher than in previous clusters 1 and 2 (95.0%). Anti-delirium measures were almost absent. This cluster reflects patients experiencing profound respiratory distress requiring aggressive ventilation strategies and prolonged sedative use.

Cluster 4 (“late severe ARDS,” n = 475) represents the most severely ill population. Patients exhibited the highest median SAPS II scores (49.4), suggesting substantial physiological derangement. All patients were invasively ventilated (100%) with a moderate proportion of prone positioning (26.9%), and vasopressor use on day 1 was the most frequent (74.3%). Although there was a higher proportion of patients with lower sedation than cluster 3 (RASS 0 2.5%), sedation practices were intensive: almost all patients received opioids (94.1%), and midazolam use was dominant (92.0%), with only a minority sedated with propofol (15.8%). Despite profound sedation, anti-delirium interventions were slightly more available compared to Cluster 3: 14.1% of patients had in-person family visits, which may be in relation to the lower use of prone positioning, which allows for sedation windows. This group had the highest number of patients with visual or hearing impairment, and few patients with alternative admission diagnosis (convulsive status n = 1, impaired level of consciousness n = 1, postoperative surveillance n = 1).

### Relation of clusters to outcomes

Outcome information was not included in the clustering process. Interestingly, we found no significant differences in mortality at 28 days or survival days among the 4 identified clusters, with survival very high in all groups, ranging from 63 to 76%. We also found no differences in hospital or ICU length of stay (Table 2).

**Table 2:** Distribution of outcome variables in ABD clusters in the COVID-D database

	Cluster 1 (n = 335)	Cluster 2 (n = 508)	Cluster 3 (n = 161)	Cluster 4 (n = 475)	p value
Neurological status (%|x, CI 95%) (until day 21, discharge, or death)					
ABD (Delirium or coma) (%) (Delirium or coma at some point during the 21-day study period)	100 (100–100)	100 (100–100)	100 (100–100)	100 (100–100)	NA
Duration of ABD (days)	10.2 (9.51–10.88)	12.2 (11.64–12.76)	12.49 (11.6–13.38)	13.3 (12.77–13.83)	0***
Delirium (%) (presence of delirium at some point during the 21-day study period)	67.46 (62.45–72.48)	65.94 (61.82–70.07)	55.28 (47.6–62.96)	62.11 (57.74–66.47)	0.0349***
Duration of delirium (days) (only exposed)	4.13 (3.66–4.6) NA: 32.54%	5.18 (4.75–5.6) NA: 34.06%	3.72 (3.21–4.23) NA: 44.72%	4.13 (3.79–4.46) NA: 37.89%	8e-04***
Duration of delirium (days)	2.79 (2.41–3.16)	3.41 (3.06–3.77)	2.06 (1.66–2.46)	2.56 (2.29–2.84)	0.0032***
Hyperactive delirium (%) (Ever hyperactive delirium at some point during the 21-day study period)	42.41 (35.4–49.42) NA: 42.99%	54.39 (48.72–60.07) NA: 41.73%	71.67 (60.26–83.07) NA: 62.73%	48.34 (41.6–55.08) NA: 55.58%	5e-04***
Duration of hyperactive delirium for those that only had hyperactive delirium (days) (only exposed)	2.28 (1.88–2.69) NA: 75.82%	3.33 (2.95–3.71) NA: 68.31%	2.51 (1.99–3.04) NA: 73.29%	2.75 (2.29–3.2) NA: 78.53%	0.0015***
Hypoactive delirium (%) (Ever hypoactive delirium at some point during the 21-day study period)	45.55 (38.49–52.61) NA: 42.99%	41.22 (35.61–46.82) NA: 41.73%	28.33 (16.93–39.74) NA: 62.73%	45.97 (39.25–52.7) NA: 55.58%	0.0756
Duration of hypoactive delirium for those that only had hyperactive delirium (days) (only exposed)	3.39 (2.73–4.05) NA: 74.03%	3.54 (2.99–4.09) NA: 75.98%	2.65 (1.8–3.5) NA: 89.44%	2.52 (2.1–2.93) NA: 79.58%	0.0446***
Coma (%) (Ever comatose at some point during the 21-day study period)	87.16 (83.58–90.75)	97.05 (95.58–98.52)	100 (100–100)	100 (100–100)	0***
Duration of coma (days) (in the 21-day study period, or until discharge or death)	8.21 (7.55–8.88)	9.87 (9.32–10.41)	10.65 (9.81–11.48)	11.2 (10.69–11.72)	0***
Persistent coma (%) (persistently comatose until day 21 or death)	0.68 (-0.26–1.63) NA: 12.84%	19.68 (16.17–23.18) NA: 2.95%	27.33 (20.45–34.21) NA: 0%	24.21 (20.36–28.06) NA: 0%	0***
Delirium-free and coma-free (DFCF) days (in the 21-day study period, or until discharge or death)	7.66 (6.95–8.37)	5.73 (5.16–6.3)	5.43 (4.54–6.32)	4.74 (4.25–5.23)	0***
Ventilatory support (%|x, CI 95%) (until day 28, discharge, or death)					
Mechanical ventilation (MV) (%)	89.55 (86.28–92.83)	99.41 (98.74–100.08)	98.76 (97.05–100.47)	99.37 (98.66–100.08)	0***
Invasive mechanical ventilation (%)	86.27 (82.58–89.95)	99.41 (98.74–100.08)	100 (100–100)	100 (100–100)	0***
Mechanical ventilation duration	13.11 (12.12–14.11)	15.22 (14.48–15.96)	16.01 (14.69–17.33)	16.08 (15.33–16.83)	0***
28-Day Ventilator Free Days	9.7 (8.64–10.77)	7.71 (6.92–8.5)	7.24 (5.93–8.55)	7.27 (6.51–8.02)	0***
Survival (%|x, CI 95%)					
Survival (days)^†^	17.86 (17.26–18.45)	17.93 (17.44–18.42)	17.93 (17.03–18.82)	18.04 (17.53–18.55)	0.717
28-day mortality (%)	32.33 (27.29–37.37) NA: 1.19%	34 (29.85–38.15) NA: 1.57%	30.82 (23.64–37.99) NA: 1.24%	31.22 (26.98–35.47) NA: 3.58%	0.7875
Days to death in the deceased	12.49 (11.29–13.68) NA: 68.06%	13.04 (12.06–14.01) NA: 66.54%	12.49 (10.4–14.58) NA: 69.57%	12.6 (11.44–13.76) NA: 69.89%	0.7005
Length of stay (%|x, CI 95%)					
ICU length of stay (days)	16.7 (15.8–17.59)	17.11 (16.38–17.84)	17.87 (16.54–19.2)	17.9 (17.16–18.63)	0.1542
Hospital length of stay (days)	23.69 (22.52–24.85)	24.27 (23.25–25.3)	25.09 (23.44–26.73)	25.59 (24.46–26.71)	0.2594
Length of stay for patients discharged alive	17.38 (16.02–18.75) NA: 75.82%	18.76 (17.62–19.9) NA: 77.95%	21.81 (20.32–23.3) NA: 80.12%	19.8 (18.82–20.78) NA: 76.63%	0.0022***
Status at 28 days (%|x, CI 95%)					
Alive and still in the hospital on day 28	43.88 (38.57–49.19)	44.69 (40.36–49.01)	49.69 (41.97–57.41)	46.53 (42.04–51.01)	0.6108

Data regarding neurological status were collected during the first 21 days, or until death or discharge, whatever happened first. Ventilator length, survival and length of stay were collected for 28 days. ^†^Survival is defined as the mean number of days alive within the 28-day follow-up period. Survivors were assigned a value of 28; non-survivors contributed their actual number of days alive. * p < 0.05, ** p < 0.01, *** p < 0.001

Cluster 1 (“mild respiratory failure”) exhibited a shorter duration of delirium and coma compared to the other groups, with 7.66 DFCF days on average and virtually no persistent coma (0.01%). Delirium affected 67.5% of patients and had a shorter duration (4.13 days among exposed) in this cluster. Of these, 42% had at least one hyperactive delirium assessment, while 46% had at least one hypoactive delirium assessment; some patients may have experienced a mixed subtype. Coma was less frequent than in the rest of clusters (87.2%) and shorter in duration (8.21 days). Mechanical ventilation was used in 89.6% of cases, mostly invasive (86.3%), but for shorter durations (13.1 days ventilation; 9.7 ventilator-free days). This cluster represents a moderate severity trajectory with a relatively favorable neurocognitive profile.

Cluster 2 (“moderate ARDS”) begins with milder symptoms but progresses toward critical illness and deeper sedation. Delirium affects nearly the same proportion of patients as cluster 1 (65.9%), but shows the longest duration of all clusters (5.18 days). Interestingly, it has the longest duration of hyperactive delirium (3.3 days), probably in line with respiratory worsening. Mechanical ventilation is needed for 99.4% of patients, mostly invasive; therefore, coma is seen in 97% of cases for 1.5 days longer (9.87 days) than cluster 1, with minimal persistent coma. DFCF days fall significantly in this cluster (5.73 days).

Cluster 3 (“early severe ARDS”) is defined by a global need for invasive mechanical ventilation and prone position. Interestingly, delirium was less prevalent in this group, affecting only 55.3% of patients. Among those with delirium, 72% had at least one hyperactive delirium assessment and 28% had at least one hypoactive delirium assessment; this is probably in relation to the high prevalence of coma (100%) and its longer duration (10.7 days). Notably, 27.3% of patients in this group experience persistent coma. It has less DFCF days than the previous cluster (5.43 days).

Cluster 4 (“late severe ARDS”) represents the most critically ill group, often affecting more severe patients at admission who require the longest durations of sedation and support; however, they seem to differ from 3 in the fact that respiratory deterioration takes place later during admission, since most of them do not require prone positioning on day 1. Delirium is present in 62.1% of patients, lasting on average 4.1 days, with an equilibrium between hypo and hyperactive subtypes. All patients experience coma, with the longest duration (11.2 days), and there is a high incidence of persistent coma (24.2%), in relation to mechanical ventilation. These patients experience the longest ICU stays (17.9 days) and hospital stays (25.6 days). DFCF days are the lowest of all clusters (4.74 days) (Fig. 4).

The preliminary analysis of the entire cohort confirmed that patients without ABD clustered consistently into a single group (Tables S6 and S7 and Figure S2 in the Supplement). In contrast, Clusters 2 and 3 were characterized by a 100% incidence of both acute brain dysfunction and mechanical ventilation, showing a profound neurocognitive burden with significantly fewer delirium- and coma-free days (mean 4.9–6.0 vs. 13.0 days) and a higher incidence of persistent coma (23% vs. 1%). Notably, while 28-day mortality was significantly lower in the non-ABD cluster (23% vs. 30–35%), survival remained uniformly low across the most severe ABD phenotypes, justifying our subsequent focused analysis on the heterogeneity within the 1,631 patients with brain dysfunction (Tables S6 and S7 in the Supplement, Figure S2).

## Discussion

In our pilot study, we identified four distinct clinical clusters of critically ill COVID-19 patients with ABD using an unsupervised machine learning approach. Using demographic and clinical variables available at ICU admission, we identified four distinct patient clusters that showed meaningful differences in the type and duration of ABD but did not differ in 28-day mortality or length of stay.

In this analysis, we found differences in the incidence of both coma and delirium among the four clusters. Persistent coma, defined as coma until death or day 21, is a defining feature of Clusters 3 and 4. Prolonged unconsciousness after COVID-19 is a widely studied complication [35], with several factors such as long-duration delirium, severe hypoxic encephalopathy [36], concomitant pathologies such as stroke [37], or even bleeding or coagulation disorders [38]. Neurocognitive recovery, as measured by DFCF days, showed a stepwise decline from Cluster 1 to Cluster 4.

Delirium incidence ranged from 55 to 67% across clusters, being slightly higher in Clusters 1 and 2. Clusters 3 and 4 showed a trend towards lower prevalence, possibly due to a higher incidence of coma and persistent coma, limiting delirium assessment. Sedative choice may have influenced these patterns. Benzodiazepines were more frequently used in Clusters 3 and 4, whereas propofol and dexmedetomidine were more common in Cluster 2. While benzodiazepines are well-documented risk factors for delirium [23], these patterns also suggest confounding by illness severity, as benzodiazepines were frequently reserved for patients with greater hemodynamic instability or those harder to sedate [39, 40]. Patients in Cluster 3, often hemodynamically unstable, may have received midazolam for its cardiovascular profile despite its association with higher delirium risk. Length of mechanical ventilation, another risk factor for delirium [9], which may contribute from Cluster 2 onwards and particularly in Cluster 4, which had the longest ventilation duration. The difference in duration of delirium may also influence clinical outcome; in previous works, patients with rapidly reversible sedation-related delirium show shorter lengths of stay and survival at 1-year compared to those with persistent delirium [41]. In this context, it is important to note that the COVID-D database recorded the daily presence of delirium rather than the exact duration of each episode. Future studies would benefit from more frequent, real-time delirium screening [11, 12].

To our knowledge, this is among the first studies to identify clusters in critically ill COVID-19 patients with ABD using unsupervised clustering at ICU admission. While some previous studies focused on delirium [20], our approach integrates both coma and delirium, offering a more complete view of neurological dysfunction. This is important given the high prevalence of deep sedation and coma in mechanically ventilated patients, where evaluating delirium alone may provide an incomplete representation of neurocognitive status [42]. Furthermore, the fact that these clusters differentiate neurocognitive trajectories without stratifying mortality suggests they capture specific brain vulnerability rather than just overall disease severity. Previous clustering studies have used variables gathered over the entire ICU stay [16, 17, 20] although this may better reflect evolving patient heterogeneity, it is less feasible for early prediction and timely clinical decision-making. In this context, future research should evaluate whether these early clusters predict outcomes such as mortality or long-term impairment, and how their performance compares to tools like the PRE-DELIRIC score [14]. Similar strategies in ARDS research have shown promising results [15].

A major strength of our study lies in the robust methodological framework, notably the use of hierarchical clustering (HC) with 1,000 bootstrap samples. Given HC’s sensitivity to early clustering decisions, this iterative approach enabled a detailed assessment of cluster stability. We tracked group assignments and centroid positions across runs and applied a binomial test to identify consistently classified patients [34]. Our approach addresses the challenge of overlapping data and contributes to improved reproducibility, offering a novel framework for identifying distinct clusters in complex clinical datasets [32, 33].

Our work has several limitations. First, the study cohort was limited to patients with COVID-19, which, while offering a clinically homogeneous sample, may restrict generalizability to other forms of critical illness or mixed ICU populations. Second, clustering was performed using only variables available on ICU admission to allow early stratification. While this enhances clinical applicability, it does not account for later interventions—such as vasopressors, corticosteroids, prone positioning, or ECMO—that may shape patient trajectories. Furthermore, the 'Day 1' window may vary in duration for each patient depending on the time of admission, which might affect the classification of early interventions. Including dynamic variables will be important in future models.

Third, the distinction between some input variables and outcomes is inherently blurred by ICU clinical reality, where sedation practices directly influence coma and delirium duration. These clusters should therefore be interpreted as 'integrated clinical phenotypes'—reflecting both the patient’s initial severity and the effects of necessary life-support measures—rather than isolated, purely biological states. Fourth, we focused on acute neurological outcomes but did not have long-term follow-up data to assess persistent cognitive deficits, recovery, or post-intensive care syndrome (PICS). We used the term ABD to encompass both delirium and coma, as it effectively summarizes early neurological impairment in the ICU. However, we acknowledge the ongoing debate around this terminology, as 'acute' may underrepresent the potential for long-term consequences [2].

Fifth, while we prioritized robustness and interpretability using bootstrapping and statistical stability analyses, a range of alternative unsupervised methods may offer complementary insights. These include model-based techniques like latent profile analysis (LPA)—the numerical-data counterpart to latent class analysis (LCA), which is suited for categorical variables and uses probabilistic classification to infer latent subgroups—, which uses probabilistic classification for numerical data; Gaussian mixture models (GMM), which assume clusters are mixtures of Gaussian distributions; and density-based methods such as HDBSCAN or OPTICS that can detect clusters of varying population density and arbitrary shapes. Future work may explore these approaches to further refine patient subphenotyping and assess the reproducibility of our findings.

Sixth, although we focused on ABD in COVID-19 patients, similar methods could be applied to broader ICU populations. In this study, our primary aim was to characterize the profiles of patients who developed delirium and/or coma; therefore, we excluded those without ABD, improving internal consistency but potentially limiting generalizability. Future research should include both ABD and non-ABD patients to assess broader applicability. Seventh, these patients were treated in the early stage of the pandemic, before major shifts in clinical management—with less use of mechanical ventilation and the introduction of immunomodulatory therapies—, which may partly explain the lack of survival differences [39]. Lastly, data collection was completed at 28 days, so potential differences beyond 28 days were not captured, limiting long-term assessment.

While machine learning holds promise for uncovering hidden patterns in critical care data, its application remains at an early stage. Clustering can aid hypothesis generation, even when subgroup distinctions are not apparent. Our findings—particularly the lack of survival differences despite well-documented relationship between ABD incidence and survival in critical care—may reflect overlapping clinical phenotypes. This underscores the need for cautious interpretation, prospective validation, and integration with supervised methods. Future work with machine-learning derived ABD clusters should be explored in cohorts with a wider spread of severity of illness and clinical outcomes.

## Conclusions

This pilot analysis based on ICU admission data from the first COVID-19 wave suggested the existence of clinically and neurologically distinct clusters among critically ill COVID-19 patients with acute brain dysfunction. We found meaningful differences in neurocognitive outcomes across clusters, particularly in the type and duration of delirium and coma, but no significant differences in survival were observed. This preliminary work may support targeted delirium prevention strategies in the ICU, but prospective studies are required to determine their clinical utility.

## Take home message

Acute brain dysfunction (ABD) in critically ill COVID-19 patients may present heterogeneously as a collection of distinct clusters. In this pilot investigation using unsupervised machine learning based on patients’ admission variables, we found four clusters characterized by differences in disease severity, sedation exposure, ventilatory support, and different degrees of ABD. One cluster of patients experienced transient delirium with minimal support while another developed deep, prolonged, iatrogenic coma under heavy doses of sedation. These exploratory findings could help inform the individualization of antidelirium strategies in the ICU, although its application in this context remains at an early stage and requires further evaluation.

## Supplementary Information

Supplementary material 1.

## Data Availability

The data that support the findings of this study are available from the authors upon reasonable request.
