# Supplementary material for: Acute brain dysfunction clusters in COVID-19: a pilot machine learning-based analysis of the COVID-D cohort
Source: Intensive Care Med Exp. 2026 Jun 8;14:69. doi: 10.1186/s40635-026-00922-4 (PMC13247105; doi:10.1186/s40635-026-00922-4)
Supplement: Supplementary file 1 — Supplementary material 1. [file 40635_2026_922_MOESM1_ESM.docx]

**Acute brain dysfunction clusters in COVID-19: a pilot machine learning-based analysis of the COVID-D cohort.**

Nekane Romero-García MD, PhD ^a, b, c †^*, Víctor Montosa-i-Micó MsC ^d†^, David Fernández-Narro MsC ^d^, Juan M. García-Gómez MsC, PhD ^d^, Luis Hurtado MD, PhD ^a, b, c^, Eduardo Passariello MD, PhD ^a, b, c^, Fabio Silvio Taccone MD, PhD ^e^, Chiara Robba MD, PhD ^f,g^, Rameela Raman MsC, PhD ^h,j^, Onur M. Orun MsC, PhD ^h,j^, Pratik Pandharipande MD, PhD ^h,i^, Brenda T. Pun MD, PhD ^h,k^, E. Wesley Ely MD, PhD ^h, k, L^, Rafael Badenes MD, PhD ^a, b, c †^, Carlos Sáez MsC, PhD ^d†^.

**SUPPLEMENTARY MATERIALS**

**INDEX**

1. **Statistical analysis**
   1. **Hierarchical clustering.**
   2. **Cluster quality assessment.**
   3. **Patient group harmonization**
2. **Tables and Figures.**
   1. **Figure S1.** Progressive Matching and Refinement of Cluster Centroids Across Experiments
   2. **Table S1.** Summary of input variables in the study population.
   3. **Table S2.** Summary of outcome variables in the study population.
   4. **Table S3.** ICU admission diagnosis in the different endotypes
   5. **Table S4.** Distribution of input variables in ABD endotypes in the COVID-D database.
   6. **Table S5.** Distribution of output variables in ABD endotypes in the COVID-D database
   7. **Table S6.** Distribution of input variables in ABD endotypes in the COVID-D database including ABD and non-ABD patients.
   8. **Table S7.** Distribution of output variables in ABD endotypes in the COVID-D database including ABD and non-ABD patients.
   9. **Figure S2**. Three-dimensional PCA plot for cluster distribution for database including ABD and non-ABD patients.
3. **STATISTICAL ANALYSIS.**
   1. **Hierarchical clustering.**

The clustering process began by retaining 20%, 40%, 50%, 60%, or 80% of the explanatory variance through PCA. Hotelling's T-squared distribution (T²) was then applied to filter out outliers. Hierarchical clustering (HC) was conducted on 1000 boostrap samples to ensure statistical robustness. In each iteration, 80% of the sample was selected using random seeds. The outcomes of each patient's group assignment and the centroid data for each group in every experiment were recorded. The robustness analysis of HC results was tested for k values of 3, 4, 5, and 6, representing the number of endotypes we aimed to identify within our sample. This range was selected as it falls within the margins of obtaining clear and clinically meaningful endotypes.

- 1. **Cluster quality assessment**

Cluster endotypes quality was further evaluated using both the general robustness analysis value (R) and the Silhouette Mean Index (S). The R metric calculated the percentage of robust patients in each endotype relative to the highest percentage, while the S metric measured cluster cohesion and separation. Heatmaps of R and S values across different k values and PCA variances were used to assess clustering performance.

The following parameters were used for cluster quality assessment:

**A.** General robustness analysis value (R) formula for a given k and variability amount from PCA, where E is the set of endotypes, Pi is the percentage of robust patients in endotype i, and Pmax is the highest percentage value of any endotype in the clustering. This parameter evaluates clustering quality by comparing the percentage of robust patients in each endotype to the best-performing endotype. It is calculated by dividing each endotype's percentage by the highest percentage value and summing these results.


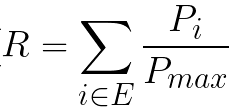


**B.** Average Silhouette Mean Index (S) for 1000 experiments formula for a given k and variability amount from PCA, where Si is the Silhouette coefficient for the i-th experiment. This measures clustering quality by assessing cohesion (within-cluster closeness) and separation (between-cluster distance). A high value indicates well-defined, separated clusters.


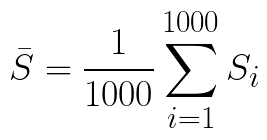


- 1. **Patient group harmonization**

Conducting robustness analysis was crucial for thoroughly understanding the sample and demonstrating the quality of our clustering. The designed process for determining robustness involved the following steps:

1. To enhance the consistency of group identification across different experiments, thereby addressing the issue of phenotypes being assigned to varying group numbers in each iteration, the following steps were implemented (Figure S1A):

Initial Comparison (Experiments 2 and 1):

- Centroids from Experiment 2 were compared to those from Experiment 1.
- The Hungarian algorithm (28) was applied to match the most similar groups between these two experiments.
- Each group from Experiment 2 was uniquely matched to a group from Experiment 1.

Progressive Refinement (Experiment 3 onwards):

- Mean centroids of groups identified in previous experiments were compared to those in subsequent experiments.
- This process refined the matching, progressively strengthening the average of similar centroids over time.
- This method made the cluster "identities" more robust with each iteration.

Final Group Assignment:

- A table indicating the frequency with which each patient was assigned to each group across all experiments was compiled.
- Each patient was assigned to the group in which they appeared most frequently across all experiments.

The decision on the choice of k and the optimal amount of variability collected was given by the results of the robustness analysis and the Silhouette coefficient assessment (Figure S1B and Figure S1C). Specific statistical analyses were conducted to evaluate the robustness within and between groups. For each patient, a binomial test was used to determine if the frequency of their most prevalent group assignment significantly exceeded the combined frequencies of all other groups. This statistical approach assessed the robustness of inter- and intragroup classifications and facilitated the identification of robust subgroups of patients within each group, thereby enhancing the precision of the clustering results.

1. **TABLES AND FIGURES.**
   1. **Figure S1.**


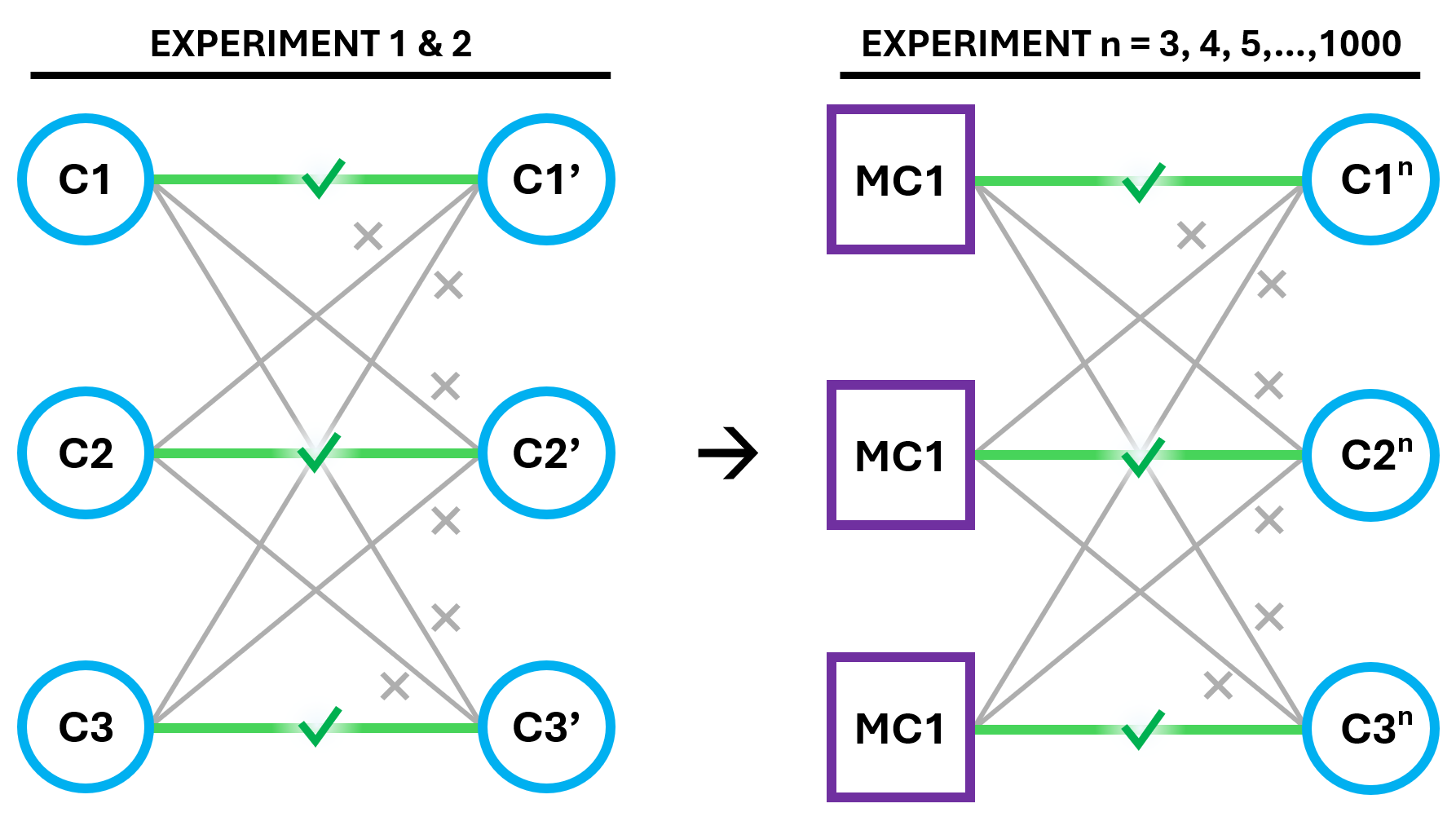


**Figure S1. Progressive Matching and Refinement of Cluster Centroids Across Experiments.** Circles represent centroids (C) from different experiments, and squares denote mean centroids (MC) from previous iterations. Green lines indicate the optimal centroid matching using the Hungarian algorithm, while gray lines represent suboptimal matches. In the left matrix, centroids from Experiment 1 are matched to those in Experiment 2. In the right matrix, from Experiment 3 onward, mean centroids (MC) are iteratively refined and matched with new centroids until n = 1000.

- 1. **Table S1.** Summary of input variables in the study population.

| **Input variables** | **Mean \| %** | **CI_95** | **Standard deviation** |
| --- | --- | --- | --- |
| Age at admission | 62.39 | 61.84 - 62.94 | 11.87 |
| Sex (Male) (%) | 28 | 0.26 - 0.30 | 0.45 |
| Hearing impairment | 0.05 | 0.04 - 0.06 | 0.21 |
| Visual impairment | 0.02 | 0.01 - 0.03 | 0.14 |
| SAPS II score | 44.77 | 44.00 - 45.54 | 16.60 |
| Active smoker | 0.09 | 0.08 - 0.10 | 0.28 |
| History of alcohol abuse | 0.04 | 0.03 - 0.05 | 0.20 |
| Hospital days before ICU | 2.99 | 2.75 - 3.23 | 5.15 |
| Charlson category 1 | 0.60 | 0.58 - 0.62 | 0.49 |
| Charlson category 2 | 0.82 | 0.80 - 0.83 | 0.39 |
| Charlson category 3 | 0.99 | 0.99 - 1.00 | 0.07 |
| Charlson category 4 | 0.99 | 0.98 - 0.99 | 0.11 |
| Type of ICU (1 medical, 2 surgical, 3 both) | 2.11 | 2.06 - 2.15 | 0.96 |
| Room_Air | 0.01 | 0.00 - 0.01 | 0.08 |
| Low flow nasal cannula or mask | 0.21 | 0.20 - 0.23 | 0.41 |
| High flow nasal cannula | 0.16 | 0.14 - 0.18 | 0.37 |
| Noninvasive mechanical ventilation | 0.08 | 0.07 - 0.10 | 0.28 |
| Invasive mechanical ventilation | 0.78 | 0.76 - 0.80 | 0.41 |
| Supine position | 0.89 | 0.87 - 0.90 | 0.32 |
| Prone position | 0.31 | 0.28 - 0.33 | 0.46 |
| Vasopressor use | 0.54 | 0.51 - 0.56 | 0.50 |
| Opioids | 0.71 | 0.69 - 0.73 | 0.45 |
| Antipsychotics | 0.02 | 0.01 - 0.02 | 0.12 |
| Anxiolythics or hypnotics | 0.08 | 0.06 - 0.09 | 0.26 |
| Propofol | 0.40 | 0.38 - 0.42 | 0.49 |
| Midazolam | 0.45 | 0.43 - 0.47 | 0.50 |
| Dexmedetomidine | 0.03 | 0.02 - 0.04 | 0.17 |
| Sevoflurane | 0.01 | 0.00 - 0.01 | 0.08 |
| Lorazepam | 0.00 | 0.00 - 0.00 | 0.04 |
| Clonidine | 0.01 | 0.00 - 0.01 | 0.08 |
| Ketamine | 0.01 | 0.01 - 0.02 | 0.10 |
| Restrained to bed | 0.17 | 0.15 - 0.19 | 0.37 |
| Access to digital devices ( phone, tablet…) | 0.13 | 0.12 - 0.15 | 0.34 |
| In-person visits | 0.06 | 0.05 - 0.08 | 0.25 |
| Virtual contact | 0.07 | 0.06 - 0.08 | 0.25 |
| Light sedation (RASS -1 or -2) | 0.05 | 0.04 - 0.06 | 0.22 |
| Deep sedation (RASS -3 to -4) | 0.03 | 0.02 - 0.04 | 0.18 |
| Agitated (RASS >0) | 0.02 | 0.02 - 0.03 | 0.14 |

- 1. **Table S2.** Summary of outcome variables in the study population.

| **Variable** | **Mean \| %** | **CI_95** | **SD** |
| --- | --- | --- | --- |
| ABD (Delirium or coma) (%) | 1.00 | 1.00 - 1.00 | 0.00 |
| Duration of ABD (days) | 12.22 | 11.92 - 12.51 | 6.33 |
| Delirium (%) | 0.65 | 0.62 - 0.67 | 0.48 |
| Duration of delirium (days) (only exposed) | 4.47 | 4.27 - 4.68 | 3.49 |
| Duration of delirium (days) | 2.89 | 2.72 - 3.05 | 3.53 |
| Duration of hyperactive delirium (days) (only exposed) | 0.52 | 0.49 - 0.55 | 0.50 |
| Hyperdelirium_duration_exp | 2.85 | 2.65 - 3.05 | 2.21 |
| Hypoactive delirium (%) | 0.42 | 0.39 - 0.45 | 0.49 |
| Duration of hypoactive delirium (days) (only exposed) | 3.07 | 2.80 - 3.35 | 2.75 |
| Coma (%) | 0.96 | 0.95 - 0.97 | 0.20 |
| Duration of coma (days) | 10.05 | 9.77 - 10.33 | 6.12 |
| Days free from ABD | 5.67 | 5.38 - 5.96 | 6.19 |
| Persistent coma (%) | 0.18 | 0.17 - 0.20 | 0.39 |
| Mechanical ventilation (MV) (%) | 0.97 | 0.96 - 0.98 | 0.17 |
| Invasive mechanical ventilation (%) | 0.96 | 0.96 - 0.97 | 0.18 |
| Mechanical ventilation duration | 15.19 | 14.78 - 15.60 | 8.77 |
| Free days from mechanical ventilation | 7.81 | 7.39 - 8.23 | 9.03 |
| ABD duration (days) | 12.22 | 11.92 - 12.51 | 6.33 |
| Survival (days) | 17.89 | 17.62 - 18.15 | 5.68 |
| 28-day mortality (%) | 0.33 | 0.31 - 0.35 | 0.47 |
| Days to death in the deceased | 12.68 | 12.13 - 13.23 | 6.75 |
| Days until Hospital Discharge | 18.98 | 18.40 - 19.56 | 5.91 |
| enr_status_days_dead | 12.68 | 12.13 - 13.23 | 6.75 |
| ICU length of stay (days) | 17.38 | 16.99 - 17.78 | 8.41 |
| Index_length_of_stay | 17.23 | 16.84 - 17.62 | 8.42 |
| Hospital length of stay | 24.86 | 24.29 - 25.43 | 12.28 |
| Race_White or Caucasian | 0.77 | 0.75 - 0.79 | 0.42 |
| Race_Black or African Descent | 0.07 | 0.06 - 0.08 | 0.25 |
| Race_Other | 0.06 | 0.05 - 0.07 | 0.24 |
| Race_Asian | 0.01 | 0.01 - 0.02 | 0.11 |
| Race_American Indian or Alaska Native | 0.06 | 0.05 - 0.08 | 0.25 |
| Race_Not Reported | 0.02 | 0.02 - 0.03 | 0.15 |
| Alive and still in the hospital on day 28 | 0.45 | 0.43 - 0.48 | 0.50 |
| Died in the index ICU before day 28 | 0.31 | 0.29 - 0.33 | 0.46 |
| Discharged alive and did not die in 28 days | 0.20 | 0.18 - 0.22 | 0.40 |
| Died in hospital ward or another ICU (discharged from index ICU) before day 28 | 0.01 | 0.01 - 0.02 | 0.11 |
| Discharged alive before day 28 (death unknown) | 0.02 | 0.02 - 0.03 | 0.15 |
| Discharged alive and died in 28 days | 0.00 | 0.00 - 0.00 | 0.02 |

- 1. **Table S3.** ICU admission diagnosis in the different clusters.

|  | **Cluster 1**  **(n = 335)** | **Cluster 2 (n = 508)** | **Cluster 3 (n = 161)** | **Cluster 4**  **(n = 475)** |
| --- | --- | --- | --- | --- |
| SARS-CoV-2 | **331** | 498 | **161** | 472 |
| Other | 4  - Acute myocardial infarction (1)  - Cardiogenic shock (1)  - Impaired level of consciousness (not specified) (1)  - Postoperative surveillance (not specified) (1) | - Chronic liver cirrhosis (1)  - Cardiac surgery (1)  - Seizures (2)  - Alcoholic deprivation (1)  - Traumatic brain injury (1)  - Cardiac arrest (1)  - Hyperkalemia (missed dialysis) (1) | 0 | - Convulsive status (1)  - Postoperative surveillance (not specified) (1)  - Impaired level of consciousness (not specified) (1) |

**Table S4.** Distribution of input variables in ABD endotypes in the COVID-D database.

|  | **Cluster 1**  **(n = 335)** | **Cluster 2 (n = 508)** | **Cluster 3 (n = 161)** | **Cluster 4**  **(n = 475)** | **p value** |  |
| --- | --- | --- | --- | --- | --- | --- |
| **Epidemiological data (%\|x, CI 95%)** | | | | | | |
| Age at admission | 63.14 (61.84-64.45) | 62.42 (61.41-63.43) | **59.88 (57.94-61.81)** | 62.67 (61.59-63.75) | 0.034*** |  |
| Sex | 27.46 (22.68-32.24) | 29.53 (25.56-33.49) | 31.06 (23.91-38.2) | 29.05 (24.97-33.14) | 0.8544 |  |
| Hearing impairment | 4.18 (2.04-6.32) | 0 (0-0) | 0.62 (-0.59-1.83) | 6.74 (4.48-8.99) | 0*** |  |
| Visual impairment | 0.9 (-0.11-1.9) | 0 (0-0) | 0 (0-0) | 2.53 (1.12-3.94) | 5e-04*** |  |
| **Previous dease (%\|x, CI 95%)** | | | | | | |
| SAPS II score | **36.93 (35.42-38.44)** | 44.31 (43.01-45.61) | 44.47 (41.94-46.99) | **49.42 (47.84-51.01)** | 0*** |  |
| Active smoker | **9.85 (6.66-13.04)** | 5.71 (3.69-7.73) | 6.83 (2.94-10.73) | 1.89 (0.67-3.12) | 0*** |  |
| History of alcohol abuse | **3.28 (1.38-5.19)** | 1.77 (0.62-2.92) | **4.35 (1.2-7.5)** | 0.84 (0.02-1.66) | 0.0171*** |  |
| Days in hospital before ICU admission | 2.67 (2.23-3.1) | 2.39 (2.12-2.66) | 2.83 (2.36-3.3) | **3.15 (2.73-3.56)** | 0.0153*** |  |
| Charlson category 1 | 52.24 (46.89-57.59) | 59.65 (55.38-63.91) | 69.57 (62.46-76.67) | 68 (63.81-72.19) | 0*** |  |
| Charlson category 2 | 79.1 (74.75-83.46) | 82.87 (79.6-86.15) | 85.09 (79.59-90.59) | 83.58 (80.25-86.91) | 0.2775 |  |
| Charlson category 3 | 100 (100-100) | 100 (100-100) | 100 (100-100) | 100 (100-100) | NA |  |
| Charlson category 4 | 100 (100-100) | 100 (100-100) | 100 (100-100) | 100 (100-100) | NA |  |
| **ICU details (%\|x, CI 95%)** | | | | | | |
| Type of ICU (1 medical, 2 surgical, 3 both) | 1.87 (1.76-1.98) | 1.99 (1.9-2.07) | 2.09 (1.96-2.23) | 2.29 (2.21-2.37) | 0*** |  |
| **Support (%\|x, CI 95%)** | | | | | | |
| Room_Air | 0 (0-0) | 0 (0-0) | 0 (0-0) | 0 (0-0) | NA |  |
| Low flow nasal cannula or mask | 44.18 (38.86-49.5) | 19.09 (15.68-22.51) | 2.48 (0.08-4.89) | 15.37 (12.13-18.61) | 0*** |  |
| High Flow nasal cannula | 49.55 (44.2-54.91) | 10.24 (7.6-12.87) | 1.24 (-0.47-2.95) | 2.74 (1.27-4.2) | 0*** |  |
| Noninvasive mechanical ventilation | 23.28 (18.76-27.81) | **7.68 (5.36-9.99)** | 0.62 (-0.59-1.83) | 0.21 (-0.2-0.62) | 0*** |  |
| Invasive mechanical ventilation | 2.09 (0.56-3.62) | **98.43 (97.34-99.51)** | 99.38 (98.17-100.59) | 100 (100-100) | 0*** |  |
| Supine position | 98.51 (97.21-99.81) | 100 (100-100) | **0 (0-0)** | 99.16 (98.34-99.98) | 0*** |  |
| Prone position | 5.07 (2.72-7.42) | 27.95 (24.05-31.86) | **100 (100-100)** | 26.95 (22.96-30.94) | 0*** |  |
| Vasopressors day1 | 4.78 (2.49-7.06) | 62.2 (57.99-66.42) | 68.32 (61.14-75.51) | **74.32 (70.39-78.24)** | 0*** |  |
| **Sedation on day 1 (%\|x, CI 95%)** | | | | | | |
| Opioids | 4.48 (2.26-6.69) | **86.81 (83.87-89.75)** | **95.03 (91.67-98.39)** | **94.11 (91.99-96.22)** | 0*** |  |
| Antipsychotics | 0 (0-0) | 0.98 (0.13-1.84) | 0 (0-0) | 0 (0-0) | 0.0224*** |  |
| Anxiolythics or hypnotics | **11.34 (7.95-14.74)** | 5.51 (3.53-7.5) | **6.21 (2.48-9.94)** | 2.95 (1.43-4.47) | 0*** |  |
| **Sedation drugs (%\|x, CI 95%)** | | | | | | |
| Propofol | 0.9 (-0.11-1.9) | **91.34 (88.89-93.78)** | 35.4 (28.02-42.79) | 15.79 (12.51-19.07) | 0*** |  |
| Midazolam | 0.3 (-0.29-0.88) | 15.55 (12.4-18.7) | **77.64 (71.2-84.08)** | **92 (89.56-94.44)** | 0*** |  |
| Dexmedetomidine | 1.49 (0.19-2.79) | 5.12 (3.2-7.03) | 0.62 (-0.59-1.83) | 0 (0-0) | 0*** |  |
| Sevoflurane | 0 (0-0) | 0 (0-0) | 0 (0-0) | 0 (0-0) | NA |  |
| Lorazepam | 0 (0-0) | 0 (0-0) | 0 (0-0) | 0 (0-0) | NA |  |
| Clonidine | 0 (0-0) | 0 (0-0) | 0 (0-0) | 0 (0-0) | NA |  |
| Ketamine | 0 (0-0) | 0 (0-0) | 0 (0-0) | 0 (0-0) | NA |  |
| **Anti delirium measures & Neurological (%\|x, CI 95%)** | | | | | | |
| Restrained to bed | 7.16 (4.4-9.93) | 25.98 (22.17-29.8) | 16.15 (10.46-21.83) | 11.16 (8.33-13.99) | 0*** |  |
| Access to digital devices ( phone, tablet…) | 33.43 (28.38-38.48) | 10.63 (7.95-13.31) | 1.86 (-0.23-3.95) | 1.89 (0.67-3.12) | 0*** |  |
| In-person visits (family/friends) | 6.57 (3.91-9.22) | 0.2 (-0.19-0.58) | 2.48 (0.08-4.89) | 14.11 (10.98-17.24) | 0*** |  |
| Virtual contact | 20.3 (15.99-24.61) | 1.77 (0.62-2.92) | 0.62 (-0.59-1.83) | 5.05 (3.08-7.02) | 0*** |  |
| **Sedation level on day 1(%\|x, CI 95%)** |  |  |  |  |  |  |
| No sedation (RASS 0)^†^ | 81.79 (77.66-85.92) | 3.74 (2.09-5.39) | 0.62 (-0.59-1.83) | 2.53 (1.12-3.94) | 0*** |  |
| Light sedation (RASS -1 or -2) | 12.24 (8.73-15.75) | 7.28 (5.02-9.54) | 0 (0-0) | 0 (0-0) | 0*** |  |
| Deep sedation (RASS -3 to -4) | 1.19 (0.03-2.36) | 6.3 (4.19-8.41) | 0.62 (-0.59-1.83) | 1.05 (0.13-1.97) | 0*** |  |
| Deep sedation (RASS -5)^†^ | 1.49 (0.19-2.79) | 82.28 (78.96-85.6) | 98.76 (97.05-100.47) | 96.42 (94.75-98.09) | 0*** |  |
| Agitated (RASS >1) | 3.28 (1.38-5.19) | 0.39 (-0.15-0.94) | 0 (0-0) | 0 (0-0) | 0*** |  |

Dichotomic variables are represented as relative frequencies of value = 1 (0 =“no” and 1=“yes”). For ICU type, 1=medical ICU, 2=surgical ICU, 3=mixed ICU.

† Variables “no sedation” and “coma” were excluded from the analysis due to high correlation with other variables; however, they are shown here for better interpretation of endotypes.

**Table S5.** Distribution of output variables in ABD endotypes in the COVID-D database.

|  | **Cluster 1**  **(n = 335)** | **Cluster 2**  **(n = 508)** | **Cluster 3 (n = 161)** | **Cluster 4**  **(n = 475)** | **p value** |
| --- | --- | --- | --- | --- | --- |
| **Neurological status (%\|x, CI 95%)** | | | | | |
| ABD (Delirium or coma) (%) | 100 (100-100) | 100 (100-100) | 100 (100-100) | 100 (100-100) | NA |
| Duration of ABD (days) | 10.2 (9.51-10.88) | 12.2 (11.64-12.76) | 12.49 (11.6-13.38) | 13.3 (12.77-13.83) | 0*** |
| Delirium (%) | 67.46 (62.45-72.48) | 65.94 (61.82-70.07) | 55.28 (47.6-62.96) | 62.11 (57.74-66.47) | 0.0349*** |
| Duration of delirium (days) (only exposed) | 4.13 (3.66-4.6) NA: 32.54 % | 5.18 (4.75-5.6) NA: 34.06 % | 3.72 (3.21-4.23) NA: 44.72 % | 4.13 (3.79-4.46) NA: 37.89 % | 8e-04*** |
| Duration of delirium (days) | 2.79 (2.41-3.16) | 3.41 (3.06-3.77) | 2.06 (1.66-2.46) | 2.56 (2.29-2.84) | 0.0032*** |
| Hyperactive delirium (%) | 0.42 (0.35-0.49) NA: 42.99 % | 0.54 (0.49-0.6) NA: 41.73 % | 0.72 (0.6-0.83) NA: 62.73 % | 0.48 (0.42-0.55) NA: 55.58 % | 5e-04*** |
| Duration of hyperactive delirium (days) (only exposed) | 2.28 (1.88-2.69) NA: 75.82 % | 3.33 (2.95-3.71) NA: 68.31 % | 2.51 (1.99-3.04) NA: 73.29 % | 2.75 (2.29-3.2) NA: 78.53 % | 0.0015*** |
| Hypoactive delirium (%) | 0.46 (0.38-0.53) NA: 42.99 % | 0.41 (0.36-0.47) NA: 41.73 % | 0.28 (0.17-0.4) NA: 62.73 % | 0.46 (0.39-0.53) NA: 55.58 % | 0.0756 |
| Duration of hypoactive delirium (days) (only exposed) | 3.39 (2.73-4.05) NA: 74.03 % | 3.54 (2.99-4.09) NA: 75.98 % | 2.65 (1.8-3.5) NA: 89.44 % | 2.52 (2.1-2.93) NA: 79.58 % | 0.0446*** |
| Coma (%) | 87.16 (83.58-90.75) | 97.05 (95.58-98.52) | 100 (100-100) | 100 (100-100) | 0*** |
| Duration of coma (days) | 8.21 (7.55-8.88) | 9.87 (9.32-10.41) | 10.65 (9.81-11.48) | 11.2 (10.69-11.72) | 0*** |
| Days free from delirium and coma | 7.66 (6.95-8.37) | 5.73 (5.16-6.3) | 5.43 (4.54-6.32) | 4.74 (4.25-5.23) | 0*** |
| Persistent coma (%) | 0.01 (0-0.02) NA: 12.84 % | 0.2 (0.16-0.23) NA: 2.95 % | 27.33 (20.45-34.21) NA: 0 % | 24.21 (20.36-28.06) NA: 0 % | 0*** |
| **Ventilatory support (%\|x, CI 95%)** | | | | | |
| Mechanical ventilation (yes/no) | 89.55 (86.28-92.83) | 99.41 (98.74-100.08) | 98.76 (97.05-100.47) | 99.37 (98.66-100.08) | 0*** |
| Invasive mechanical ventilation (yes/no) | 86.27 (82.58-89.95) | 99.41 (98.74-100.08) | 100 (100-100) | 100 (100-100) | 0*** |
| Mechanical ventilation duration (days) | 13.11 (12.12-14.11) | 15.22 (14.48-15.96) | 16.01 (14.69-17.33) | 16.08 (15.33-16.83) | 0*** |
| Mechanical_ventilation_free_days | 9.7 (8.64-10.77) | 7.71 (6.92-8.5) | 7.24 (5.93-8.55) | 7.27 (6.51-8.02) | 0*** |
| **Survival (%\|x, CI 95%)** |  |  |  |  |  |
| Delirium_or_coma_duration_all | 10.2 (9.51-10.88) | 12.2 (11.64-12.76) | 12.49 (11.6-13.38) | 13.3 (12.77-13.83) | 0*** |
| Survival | 17.86 (17.26-18.45) | 17.93 (17.44-18.42) | 17.93 (17.03-18.82) | 18.04 (17.53-18.55) | 0.717 |
| 28-day mortality (%) | 0.32 (0.27-0.37) NA: 1.19 % | 0.34 (0.3-0.38) NA: 1.57 % | 0.31 (0.24-0.38) NA: 1.24 % | 0.31 (0.27-0.35) NA: 3.58 % | 0.7875 |
| Days to death in the deceased | 12.49 (11.29-13.68) NA: 68.06 % | 13.04 (12.06-14.01) NA: 66.54 % | 12.49 (10.4-14.58) NA: 69.57 % | 12.6 (11.44-13.76) NA: 69.89 % | 0.7005 |
| Length of stay for alive patients | 17.38 (16.02-18.75) NA: 75.82 % | 18.76 (17.62-19.9) NA: 77.95 % | 21.81 (20.32-23.3) NA: 80.12 % | 19.8 (18.82-20.78) NA: 76.63 % | 0.0022*** |
| Length of stay for deceased patients | 12.49 (11.29-13.68) NA: 68.06 % | 13.04 (12.06-14.01) NA: 66.54 % | 12.49 (10.4-14.58) NA: 69.57 % | 12.6 (11.44-13.76) NA: 69.89 % | 0.7005 |
| **Length of stay (%\|x, CI 95%)** | | | | | |
| ICU length of stay (days) | 16.7 (15.8-17.59) | 17.11 (16.38-17.84) | 17.87 (16.54-19.2) | 17.9 (17.16-18.63) | 0.1542 |
| Hospital length of stay (days) | 23.69 (22.52-24.85) | 24.27 (23.25-25.3) | 25.09 (23.44-26.73) | 25.59 (24.46-26.71) | 0.2594 |
| **Status at 28 days (%\|x, CI 95%)** | | | | | |
| Alive and still in the hospital on day 28 | 43.88 (38.57-49.19) | 44.69 (40.36-49.01) | 49.69 (41.97-57.41) | 46.53 (42.04-51.01) | 0.6108 |
| Died in the index ICU before day 28 | 30.45 (25.52-35.38) | 31.69 (27.65-35.74) | 29.19 (22.17-36.22) | 29.05 (24.97-33.14) | 0.8221 |
| Discharged alive and did not die in 28 days | 22.99 (18.48-27.49) | 20.28 (16.78-23.77) | 18.63 (12.62-24.65) | 19.79 (16.21-23.37) | 0.6214 |
| Died in hospital ward or another ICU (discharged from index ICU) before day 28 | 1.49 (0.19-2.79) | 1.57 (0.49-2.66) | 1.24 (-0.47-2.95) | 1.05 (0.13-1.97) | 0.9027 |
| Discharged alive before day 28 (death unknown) | 1.19 (0.03-2.36) | 1.57 (0.49-2.66) | 1.24 (-0.47-2.95) | 3.58 (1.91-5.25) | 0.0535 |
| Discharged alive and died in 28 days | 0 (0-0) | 0.2 (-0.19-0.58) | 0 (0-0) | 0 (0-0) | 0.5907 |

**Table S6.** Distribution of input variables in ABD endotypes in the COVID-D database including ABD and non-ABD patients.

|  | **Cluster 1**  **(n = 602 )** | **Cluster 2**  **(n = 398 )** | **Cluster 3**  **(n = 875 )** | **P.Value** |
| --- | --- | --- | --- | --- |
| **Epidemiological data (%\|x, CI 95%)** | | | | |
| Age at admission | 61.44 (60.41-62.48) | 63.2 (62.12-64.28) | 61.46 (60.67-62.25) | 0.1569 |
| Sex | 27.57 (24-31.14) | 30.4 (25.88-34.92) | 26.74 (23.81-29.68) | 0.3975 |
| Hearing impairment | 2.33 (1.12-3.53) | 0 (0-0) | 5.71 (4.18-7.25) | 0*** |
| Visual impairment | 0.83 (0.11-1.56) | 0 (0-0) | 1.37 (0.6-2.14) | 0.0555 |
| **Previous dease (%\|x, CI 95%)** | | | | |
| SAPS II score | 33.61 (32.48-34.74) | 43.26 (41.88-44.63) | 47.38 (46.24-48.52) | 0*** |
| Active smoker | 7.97 (5.81-10.14) | 2.26 (0.8-3.72) | 8.57 (6.72-10.43) | 1e-04*** |
| History of alcohol abuse | 1.99 (0.88-3.11) | 0 (0-0) | 4.46 (3.09-5.82) | 0*** |
| Days in hospital before ICU admission | 3.05 (2.7-3.39) | 2.4 (2.08-2.72) | 2.93 (2.67-3.2) | 0.0621 |
| Charlson category 1 | 53.82 (49.84-57.8) | 57.04 (52.17-61.9) | 66.06 (62.92-69.19) | 0*** |
| Charlson category 2 | 81.56 (78.46-84.66) | 83.67 (80.04-87.3) | 84.46 (82.06-86.86) | 0.3345 |
| Charlson category 3 | 100 (100-100) | 100 (100-100) | 100 (100-100) | NA |
| Charlson category 4 | 100 (100-100) | 100 (100-100) | 100 (100-100) | NA |
| **Support (%\|x, CI 95%)** | | | | |
| Room_Air | 0 (0-0) | 0 (0-0) | 0 (0-0) | NA |
| Low flow nasal cannula or mask | 43.69 (39.73-47.65) | 20.1 (16.16-24.04) | 13.14 (10.9-15.38) | 0*** |
| High Flow nasal cannula | 47.01 (43.02-51) | 10.8 (7.75-13.85) | 4 (2.7-5.3) | 0*** |
| Noninvasive mechanical ventilation | 24.92 (21.46-28.37) | 6.28 (3.9-8.67) | 2.17 (1.21-3.14) | 0*** |
| Invasive mechanical ventilation | 1.66 (0.64-2.68) | 98.74 (97.65-99.84) | 99.66 (99.27-100.04) | 0*** |
| Supine position | 98.67 (97.76-99.59) | 99.75 (99.26-100.24) | 79.43 (76.75-82.11) | 0*** |
| Prone position | 7.81 (5.66-9.95) | 32.16 (27.57-36.75) | 41.03 (37.77-44.29) | 0*** |
| Vasopressors day1 | 4.82 (3.11-6.53) | 61.06 (56.26-65.85) | 70.86 (67.85-73.87) | 0*** |
| **Sedation on day 1 (%\|x, CI 95%)** | | | | |
| Opioids | 2.82 (1.5-4.15) | 87.19 (83.9-90.47) | 92.46 (90.71-94.21) | 0*** |
| Antipsychotics | 0 (0-0) | 0 (0-0) | 0 (0-0) | NA |
| Anxiolythics or hypnotics | 14.78 (11.95-17.62) | 5.28 (3.08-7.47) | 5.6 (4.08-7.12) | 0*** |
| **Anti delirium measures (%\|x, CI 95%)** | | | | |
| Restrained to bed | 6.64 (4.65-8.63) | 21.86 (17.8-25.92) | 15.43 (13.04-17.82) | 0*** |
| Access to digital devices ( phone, tablet…) | 36.88 (33.02-40.73) | 9.05 (6.23-11.86) | 3.54 (2.32-4.77) | 0*** |
| In-person visits | 6.31 (4.37-8.25) | 0 (0-0) | 8.8 (6.92-10.68) | 0*** |
| Virtual contact | 27.74 (24.16-31.32) | 1.26 (0.16-2.35) | 3.89 (2.61-5.17) | 0*** |

**Table S7.** Distribution of output variables in ABD endotypes in the COVID-D database including ABD and non-ABD patients.

|  | **Cluster 1**  **(n = 602 )** | **Cluster 2**  **(n = 398 )** | **Cluster 3**  **(n = 875 )** | **P.Value** |
| --- | --- | --- | --- | --- |
| **Neurological status (%\|x, CI 95%)** | | | | |
| ABD (Delirium or coma) (%) | 55.48 (51.51-59.45) | 98.24 (96.95-99.53) | 99.89 (99.66-100.11) | 0*** |
| Duration of ABD (days) | 10.27 (9.58-10.96) | 12.15 (11.52-12.79) | 13.33 (12.94-13.72) | 0*** |
| Delirium (%) | 37.04 (33.19-40.9) | 60.05 (55.24-64.86) | 62.86 (59.66-66.06) | 0*** |
| Duration of delirium (days) (only exposed) | 4.13 (3.66-4.61) | 4.78 (4.32-5.24) | 4.39 (4.13-4.65) | 0.0189*** |
| Duration of delirium (days) | 1.53 (1.29-1.77) | 2.87 (2.51-3.23) | 2.76 (2.54-2.97) | 0*** |
| Hyperactive delirium (%) | 41 (34-48) | 60 (53-67) | 54 (50-59) | 3e-04*** |
| Duration of hyperactive delirium (days) (only exposed) | 2.3 (1.88-2.72) | 3.33 (2.9-3.76) | 2.87 (2.58-3.17) | 0.002*** |
| Hypoactive delirium (%) | 48 (40-55) | 38 (31-44) | 42 (37-46) | 0.1229 |
| Duration of hypoactive delirium (days) (only exposed) | 3.34 (2.7-3.98) | 2.94 (2.44-3.43) | 2.74 (2.39-3.08) | 0.1693 |
| Coma (%) | 47.84 (43.85-51.83) | 95.98 (94.05-97.91) | 99.43 (98.93-99.93) | 0*** |
| Duration of coma (days) | 4.61 (4.11-5.11) | 9.93 (9.3-10.55) | 11.07 (10.69-11.44) | 0*** |
| Days free from delirium and coma | 13.03 (12.36-13.7) | 6 (5.33-6.67) | 4.88 (4.51-5.25) | 0*** |
| Persistent coma (%) | 1.02 (0-0.20) | 22 (18-26) | 23 (2-26) | 0*** |
| **Ventilatory support (%\|x, CI 95%)** | | | | |
| Mechanical ventilation (yes/no) | 64.12 (60.29-67.95) | 99.5 (98.8-100.19) | 99.43 (98.93-99.93) | 0*** |
| Invasive mechanical ventilation (yes/no) | 49.34 (45.34-53.33) | 99.5 (98.8-100.19) | 100 (100-100) | 0*** |
| Mechanical ventilation duration (days) | 8 (7.26-8.74) | 14.81 (13.98-15.65) | 16.49 (15.92-17.05) | 0*** |
| Mechanical_ventilation_free_days | 16.33 (15.42-17.25) | 8.1 (7.18-9.01) | 7.1 (6.55-7.65) | 0*** |
| **Survival (%\|x, CI 95%)** |  |  |  |  |
| Delirium_or_coma_duration_all | 5.7 (5.14-6.26) | 11.94 (11.29-12.58) | 13.31 (12.92-13.71) | 0*** |
| 28-day mortality (%) | 23 (19-26) | 35 (30-39) | 30 (27-33) | 1e-04*** |
| Days to death in the deceased | 11.77 (10.65-12.89) NA: 78.41 % | 13.36 (12.25-14.46) NA: 65.58 % | 12.84 (11.94-13.74) NA: 71.09 % | 0.1249 |
| Length of stay for deceased patients | 13.79 (13.09-14.49) | 18.37 (16.99-19.76) | 20.15 (19.39-20.9) | 0*** |
| **Length of stay (%\|x, CI 95%)** | | | | |
| ICU length of stay (days) | 11.97 (11.27-12.66) | 16.82 (15.99-17.65) | 18.26 (17.71-18.82) | 0*** |
| Hospital length of stay (days) | 20.97 (20.11-21.83) | 24.47 (23.27-25.67) | 25.52 (24.73-26.31) | 0*** |
| **Status at 28 days (%\|x, CI 95%)** | | | | |
| Alive and still in the hospital on day 28 | 31.4 (27.69-35.1) | 42.96 (38.1-47.83) | 49.26 (45.94-52.57) | 0*** |
| Died in the index ICU before day 28 | 18.77 (15.65-21.89) | 32.66 (28.06-37.27) | 27.89 (24.91-30.86) | 0*** |
| Discharged alive and did not die in 28 days | 42.36 (38.41-46.31) | 21.61 (17.56-25.65) | 18.4 (15.83-20.97) | 0*** |
| Died in hospital ward or another ICU (discharged from index ICU) before day 28 | 4.65 (2.97-6.33) | 1.01 (0.03-1.98) | 3.43 (2.22-4.63) | 0.0066*** |
| Discharged alive before day 28 (death unknown) | 2.66 (1.37-3.94) | 1.51 (0.31-2.7) | 1.03 (0.36-1.7) | 0.0527 |
| Discharged alive and died in 28 days | 0.17 (-0.16-0.49) | 0.25 (-0.24-0.74) | 0 (0-0) | 0.3839 |


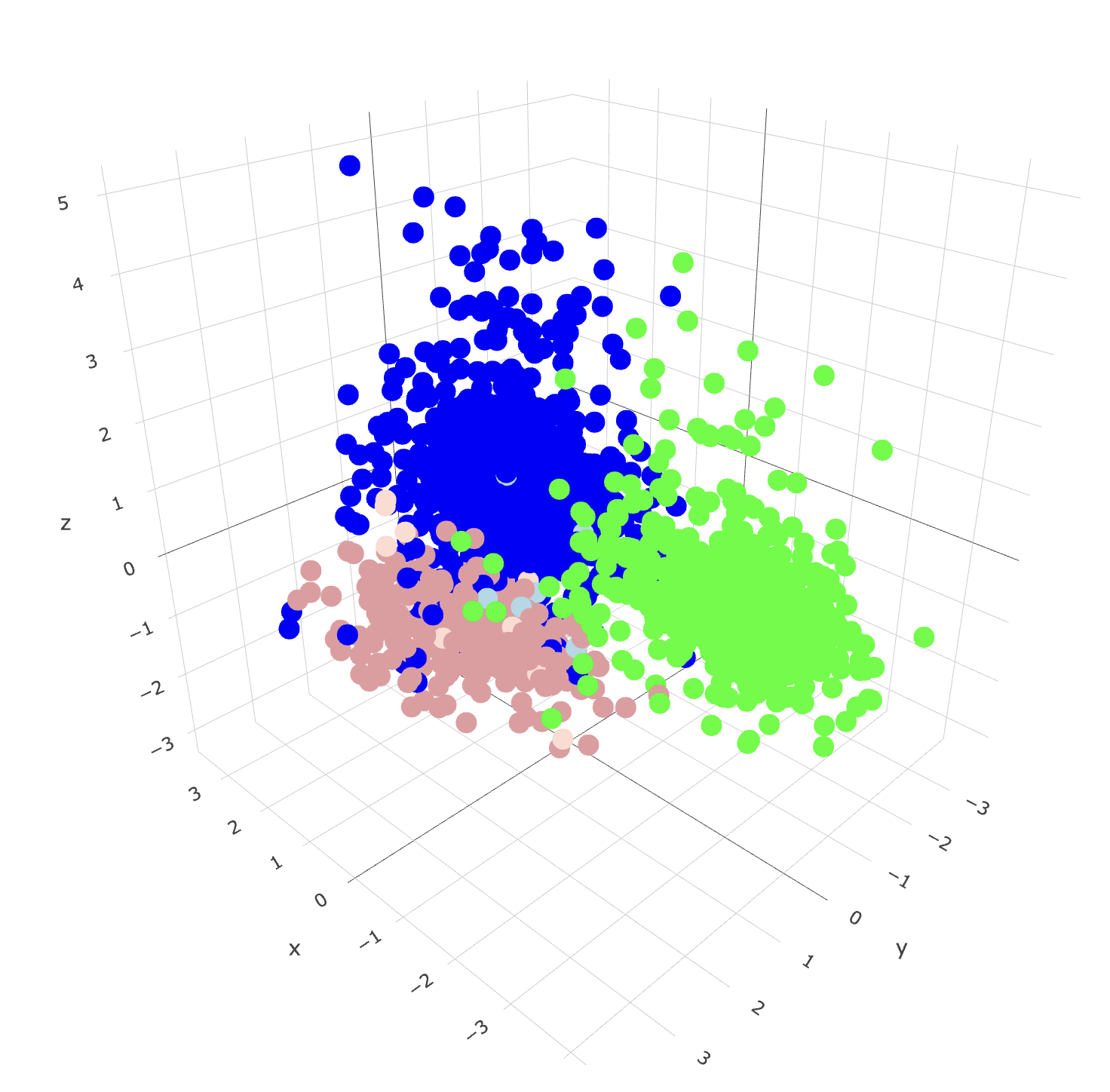


**Figure S2. Three-dimensional PCA plot for cluster distribution. 3D Scatterplot for all patients.** Dark-colored dots represent robust patients, while light-colored dots represent non-robust patients. Green represents cluster 1, pink represents cluster 2, blue represents cluster 3.
